# Supplementary material for: Cation-π interactions enabled water-stable perovskite X-ray flat mini-panel imager
Source: Nat Commun. 2024 Jan 4;15:257. doi: 10.1038/s41467-023-44644-7 (PMC10767000; doi:10.1038/s41467-023-44644-7)
Supplement: Supplementary file 1 — Supplementary Information [file 41467_2023_44644_MOESM1_ESM.pdf]

Supplementary Information for

Cation-π Interactions Enabled Water-stable Perovskite X-ray Flat Mini-panel Imager

Wanting Pan[1], Yuhong He[1], Weijun Li[1], Lulu Liu[1], Keke Guo[1], Jianglei Zhang[1], Chao Wang[2], Bao Li[1], Hu Huang[2], Junhu Zhang[1,3], Bai Yang[1,3], and Haotong Wei[1,3*]

[1]*State Key Laboratory of Supramolecular Structure and Materials, College of Chemistry, Jilin University, Changchun, 130012, P.R. China*

[2]*School of Mechanical and Aerospace Engineering, Jilin University, Changchun, Jilin, 130022, China*

[3] *Optical Functional Theranostics Joint Laboratory of Medicine and Chemistry, The First Hospital of Jilin University, Changchun, 130012 P.R. China*

[*] Correspondence to H.W. Email: hweichem@jlu.edu.cn.

Results and discussion. 3

Supplementary Figure 1 Photos of 1D single crystals and 2D single crystals........................ 3

Supplementary Figures 2-6 Dimension transition 4

Supplementary Figure 7 XRD and the Williamson-Hall (W-H) calculation for microstrain. 7

Supplementary Figure 8 Schematic diagram of the microstrain generation process............. 8

Supplementary Figure 9 Band structure study... 9

Supplementary Figure 10 Interactions analysis 10

Supplementary Figure 11 Solid-state NMR characterization 11

Supplementary Figure 12 IR measurements of TA-based perovskites................................ 12

Supplementary Figure 13 Liquid-state NMR characterization 13

Supplementary Figure 14 Morphologies study of tablets during dimension transitions 14

Supplementary Figure 15 TGA measurements.. 15

Supplementary Figure 16 Nanoindentation curves.. 15

Supplementary Figures 17, 18, 21, 22 and 23 Device sensitivity and μτ product 17

Supplementary Figure 18 water soaking stability of the F-1D tablet 18

Supplementary Figure 19 NED of the F-1D flat panel detector 19

Supplementary Figure 24 SCLC measuremnets 23

Supplementary Figure 25 response time of the detector.. 24

Supplementary Figure 26 anisotropic properties of the $TA_4Pb_3I_{10}$ perovskite material....... 25

Supplementary Figure 27 Probe card... 26

Supplementary Table 1 Anisotropic Displacement Parameters ($\overline{\mathbf{U}}_{\mathbf{eq}}$) comparision of single crystals. 27

Supplementary Methods 29

Supplementary References...33

The concentration of the Pb^{2+} in precursor solutions is 1.0 M, and the stoichiometric ratio of TA^+ to Pb^{2+} is 2:1. The sheet-like rigid 1D $TA_4Pb_3I_{10}$ single crystals (Supplementary Figure 1a) were grown from the solvent ACN at room temperature. The sheet-like 2D TA_2PbI_4 single crystal (Supplementary Figure 1b) was grown from the mixed solvent (ACN: EA=1:1) at room temperature.

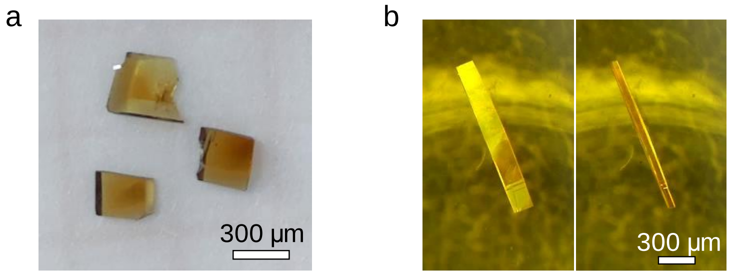

Supplementary Figure 1: Crystal image of the TA-based perovskite. a, sheet-like 1D $TA_4Pb_3I_{10}$ single crystals. **b,** sheet-like 2D TA_2PbI_4 single crystal.

In the unit cell of the 1D perovskite (Supplementary Figure 2a), three octahedrons are connected in turn by face contact and edge contact, forming the repeat unit of the inorganic part, and between these two columns of the inorganic octahedron, four TA molecules are divided into two parallel groups, with each group of the parallel molecules arranged in reverse order.

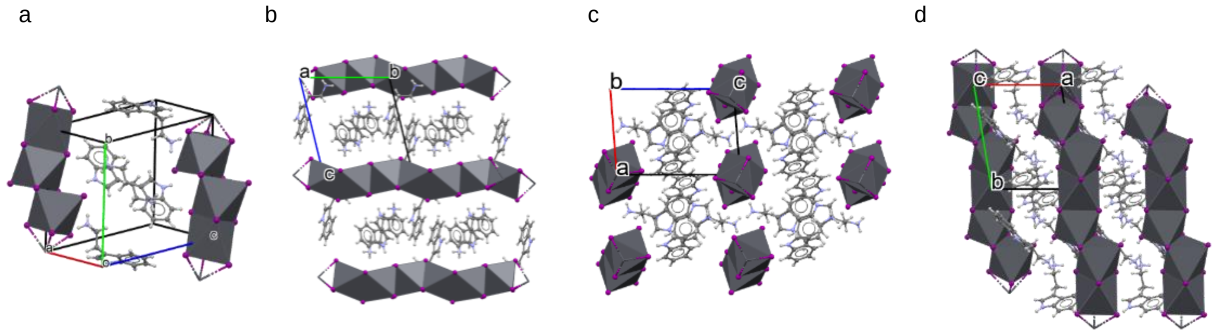

Supplementary Figure 2: Crystal structure of the 1D $TA_4Pb_3I_{10}$ perovskite. a, the unit cell of the crystal. **b-d,** the crystal structure from the side of a, b, c, respectively.

We tested the thermal annealing effect on 1D perovskite single crystals and powders. Although the noticeable color change occurred in both single crystals and powders, no dimension transitions occurred according to XRD measurements (Supplementary Figures 3a and 3b). Then we pressed the powders into the tablets and thermally annealed them at 120°C for several hours. The characteristic peaks of the 2D TA_2PbI_4 perovskite were observed when checked by XRD (Supplementary Figure 3c). To confirm the changes in the lattice structure, the microstrain during the thermal annealing process was derived by the Williamson-Hall (W-H) calculation and concluded in Supplementary Figure 3d.

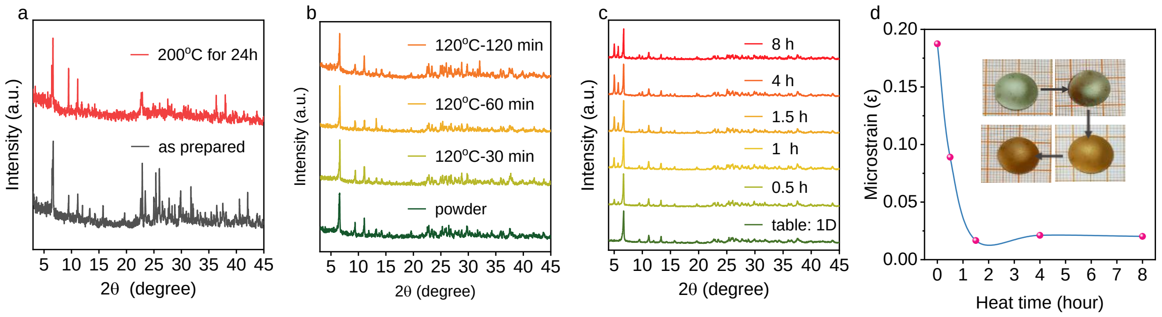

Supplementary Figure 3: Perovskite dimension transition. a, XRD spectra of 1D single crystal $TA_4Pb_3I_{10}$ before and after heat treatment. **b**, XRD spectra of 1D powder before and after heat treatment. No new peaks appeared in both the single crystal or powder cases. **c**, XRD spectra of the 1D tablet before and after heat treatment, dimension transition occurred. **d**, microstrain variation with heating time.

Williamson-Hall (W-H) calculation and fittings for the tablets with various heating times are shown in Supplementary Figure 4. Microstrain (ε) is obtained from the slope of the fitted line. As plotted in Supplementary Figure 3d, the microstrain decreased by magnitude (0.1875 to 0.020) after several hours of thermal annealing, demonstrating the microstrain relaxation process induced by thermal annealing.

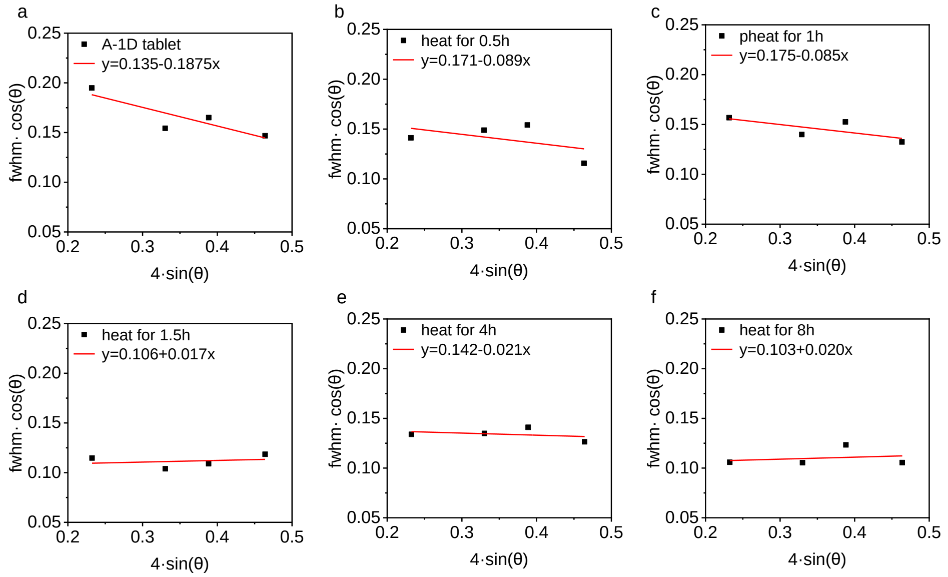

Supplementary Figure 4: The Williamson-Hall (W-H) calculation results of the tablets in different conditions. (**a-f**) represent the as-prepared state, heat-treat for 0.5 h, 1 h, 1.5 h, 4 h, and 8 h, respectively.

We conclude the XRD spectra of A-1D, A-2D and T-2D to confirm the structure change during the dimension transition process. The small-angle shift of Peaks of T-2D indicates that organic part TA^+ arranged as a more loosely packed mode than A-2D.

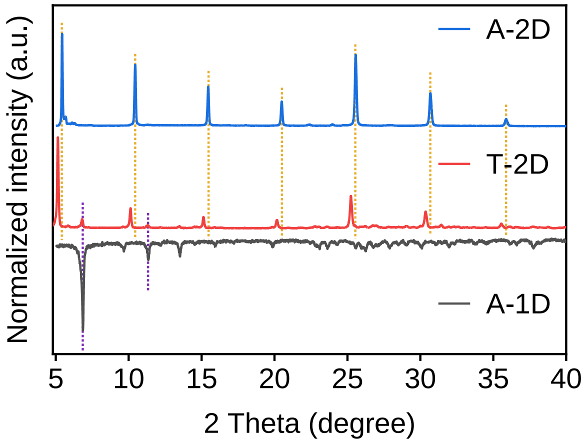

Supplementary Figure 5: XRD Characterization of A-1D, A-2D and T-2D perovskite tablets.

We found that the T-2D tablet can switch back to the T-1D/F-1D tablet upon water soaking or moisture treatment. To provide a wild transition process from 2D to 1D, we built an environment with high humidity for the dimension transition from 2D to 1D. As shown in Supplementary Figure 6, a glass culture dish filled with water was placed on a hot plate and then covered the dish with a beaker. When we raise the temperature of the hot plate, moisture will fill in the limited area, thus resulting in a high-humidity environment.

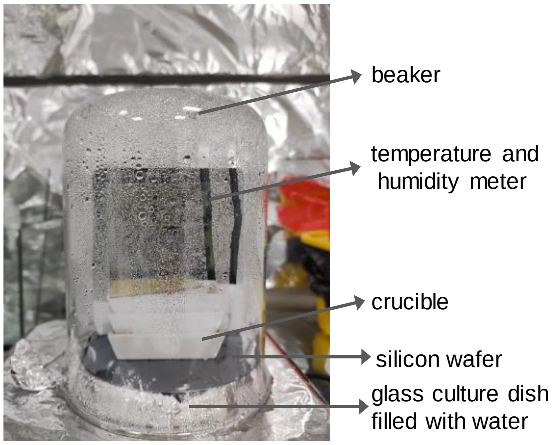

Supplementary Figure 6: High-humidity environment for dimension transition.

To confirm the changes in the lattice structure during the reversible dimension transition process, we perform the Williamson-Hall (W-H) calculation for the tablets with different states. Supplementary Figures 7a-7g represent the microstrain fitting lines of the tablets under different conditions. The microstrain increases by magnitude after tableting (0.02 to 0.17) and decreases to 0.031 after cycles of the dimension transition processes. External press tableting induced an enlarged microstrain, while the dimension transition process relaxed the microstrain.

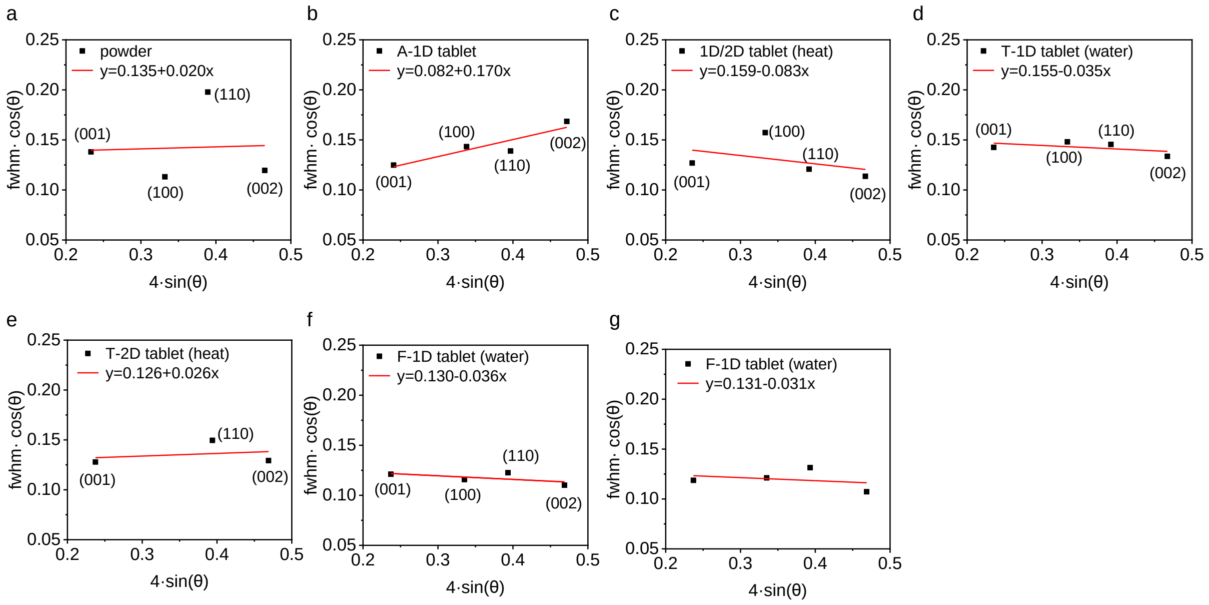

Supplementary Figure 7: The Williamson-Hall (W-H) calculation. The Williamson-Hall (W-H) calculation results of the TA-based perovskite tablets in different conditions. The slopes of the fitting lines in (**a-g**) represent the microstrain in various states.

Based on the previous XRD characterization and W-H calculation results during the tableting and transition processes. When the perovskite powder was tableted as the tablet, an enlarged microstrain in the perovskite lattice was induced. We showed this microstrain generation process as schemed in Supplementary Figure 8.

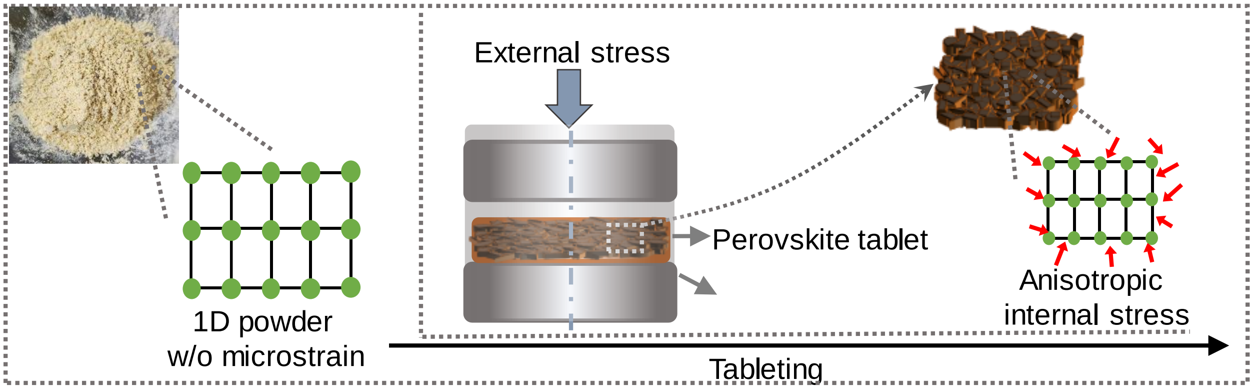

Supplementary Figure 8: The Schematic diagram of the microstrain generation process during the tableting process.

Based on the ultraviolet-visible absorption spectra (UV) (Supplementary Figure 9a) and ultraviolet photoelectron spectroscopy (UPS) measurements (Supplementary Figures 9b and 9c). The energy band structures of 1D $TA_4Pb_3I_{10}$ and 2D TA_2PbI_4 perovskites are depicted in Supplementary Figures 9d and 9e. The work function of the 1D $TA_4Pb_3I_{10}$ and the 2D TA_2PbI_4 were 4.65 eV and 4.75 eV, respectively. Precursor solutions for UV and UPS measurements were prepared by dissolving 461 mg PbI_2 and 383 mg TAI (1D) / 461 mg PbI_2, and 574 mg TAI (2D) in DMF. The spin-coating is performed at 4000 r.p.m for 30 s. For the 1D film, the annealing condition is 80°C for 20 minutes. For the 2D film, the annealing condition is 80°C for 3 minutes and 120°C for 20 minutes.

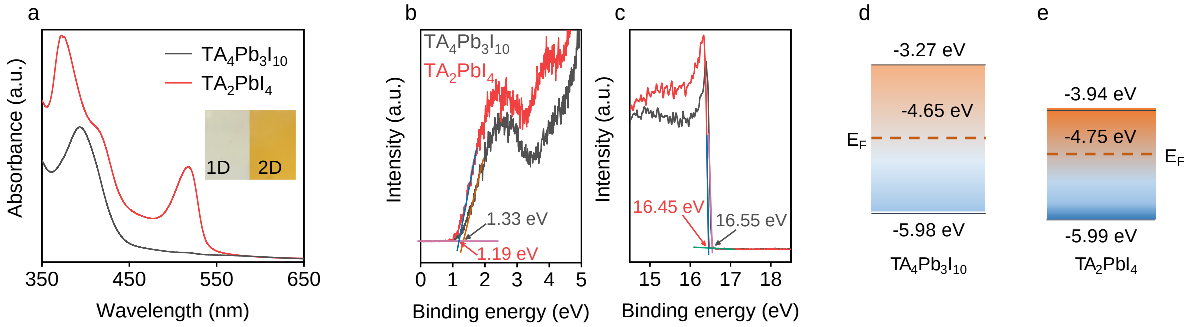

Supplementary Figure 9: Energy band structures of TA-based perovskites. a, the ultraviolet-visible absorption spectra of 1D and 2D perovskite. **b-c,** the UPS measurements. **d-e,** the energy band diagrams of the 1D and 2D perovskite.

As shown in Supplementary Figure 10a, three kinds of interactions exist in the 1D perovskite ($TA_4Pb_3I_{10}$), including halogen bonding between the Pb-I octahedrons and TA cations, π-π interactions between indole rings and cation-π interactions between pyrrole rings and the ethylamino of the neighboring TA^+, Supplementary Figures 10b and 10c exhibit the strong cation-π and π-π interactions between the closely packed TA ions, separately. And Supplementary Figure 10d shows the halogen bonding in 1D perovskite. In contrast, only halogen bonding and relatively loose π-π interactions exist in the optimized 2D structure (TA_2PbI_4) (Supplementary Figure 10e), which means a relatively loose arrangement in the 2D structure.

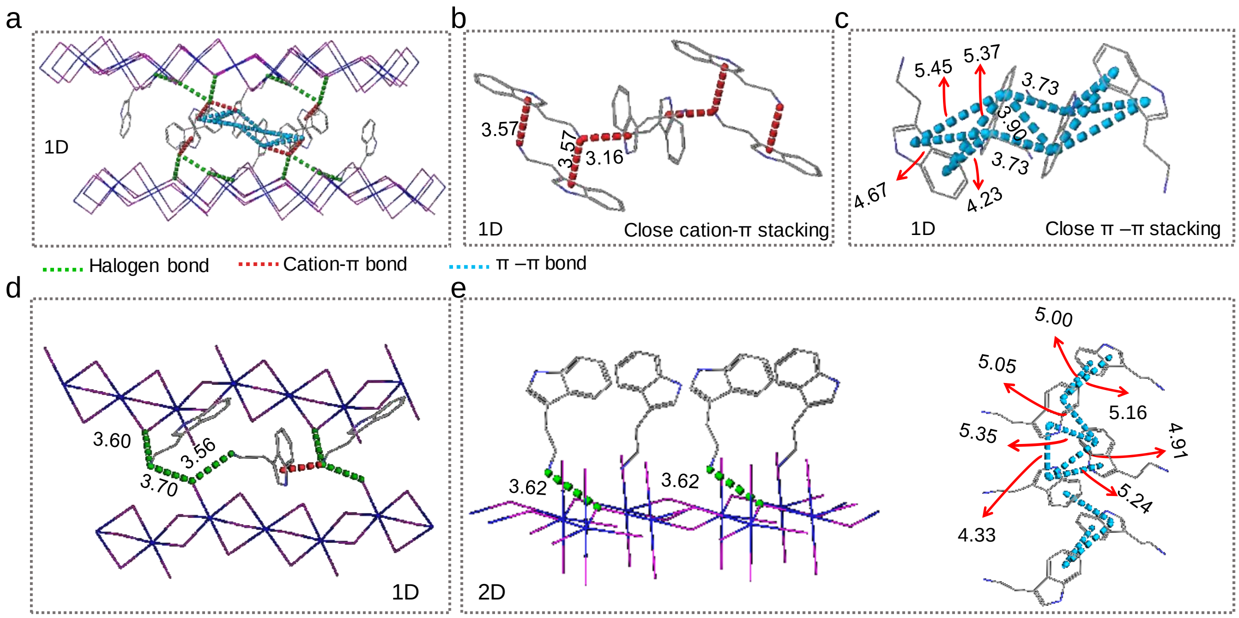

Supplementary Figure 10: Interactions between TA-based 1D and 2D perovskites. a, Visual display of interactions that exist in 1D $TA_4Pb_3I_{10}$ perovskite. **b,** Strong face-to-face cation-π in organic cations part of 1D perovskite $TA_4Pb_3I_{10}$. **c**, Strong π-π stacking in organic cations part of 1D perovskite $TA_4Pb_3I_{10}$. **d**, the halogen bonding in 1D perovskite. **e**, halogen bonding and π-π stacking between organic cations of 2D perovskite TA_2PbI_4.

Supplementary Figure 11 exhibits two-dimensional (2D) ^{13}C heteronuclear correlation (HETCOR) experiments, where the spectra along the top horizontal axis are solid-state 1D $^{13}C\{^1H\}$ CP MAS NMR, and the spectra along the left vertical axis are solid-state 1D ^1H MAS NMR. The Arabic numeral labels refer to carbons on TAI.

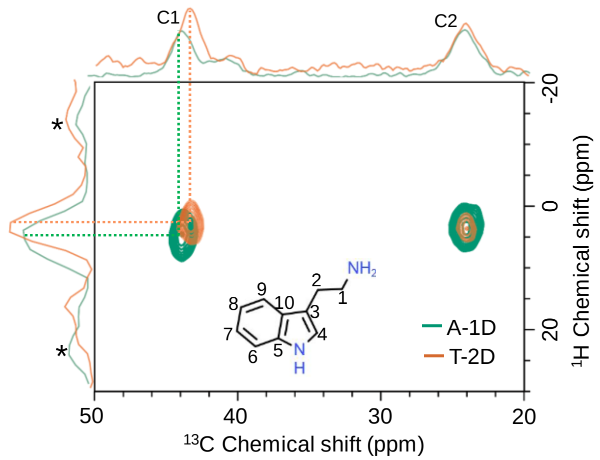

Supplementary Figure 11: Solid-state 2D $^{13}C\{^{1}H\}$ NMR spectrum. Solid-state 2D $^{13}C\{^{1}H\}$ NMR spectrum acquired from bulk TA-based perovskite with a 1D ^{13}C CP MAS NMR spectrum along the top horizontal axis and a single-pulse ^{1}H MAS spectrum along the left vertical axis. Star identifier (*) in Supplementary Figure 11 refers to the rotating sideband.

IR measurements of the reversible dimension transition process. only N-H bonds at 3375 cm^{-1} show up. After thermal annealing, the infrared vibration peak of H-N-H bonds at 3375 cm^{-1} merges, representing a more loosely packed mode in the T-2D case. And as we can see, after reversibly water treatment, the infrared vibration peak of H-N-H disappears again, indicating the recovery of the closely packing mode in the A-1D case.

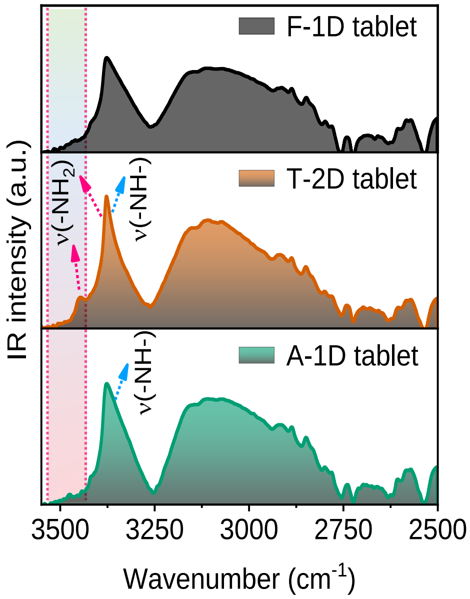

Supplementary Figure 12: IR measurements of the reversible dimension transition process.

Liquid NMR measurements were performed to confirm the structure of the TAI molecule. Based on the results, the pyrrole nitrogen is unprotonated. As depicted in Supplementary Figure 13, TAI and TAI-PbI_2 in deuterated DMSO have an utterly consistent chemical shift, indicating the entirely consistent chemical environment of hydrogens, proving that TAI molecules present a monodispersed state, whether with or without PbI_2. On the contrary, when we take the deuterated acetonitrile as the solvent to perform the same experiments, the chemical shift of the TAI-PbI_2 sample shifted compared with pure TAI molecules (Figure 3b), demonstrating that intermolecular interactions occurring in the precursor solution of TAI-PbI_2 in ACN.

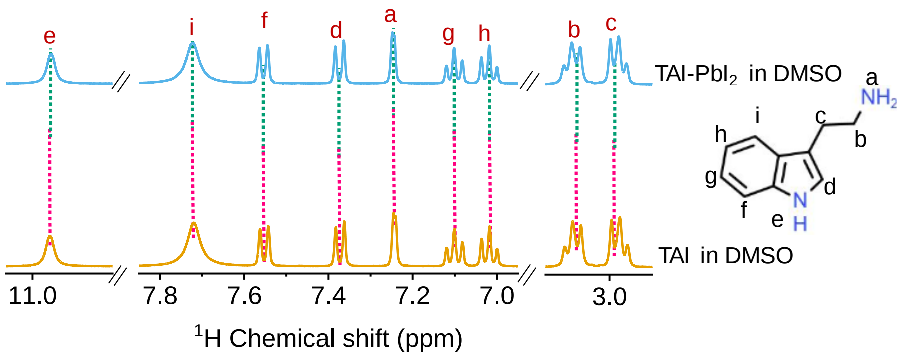

Supplementary Figure 13: Liquid NMR measurements of TAI and TAI-PbI_2 in deuterated DMSO

The morphologies evolution of the tablets are studied by SEM and AFM tests in Supplementary Figure 14. By gradually increasing the thermal annealing time, the grains gradually changed to pieces and then stabilized to be sheet layer-like morphology.

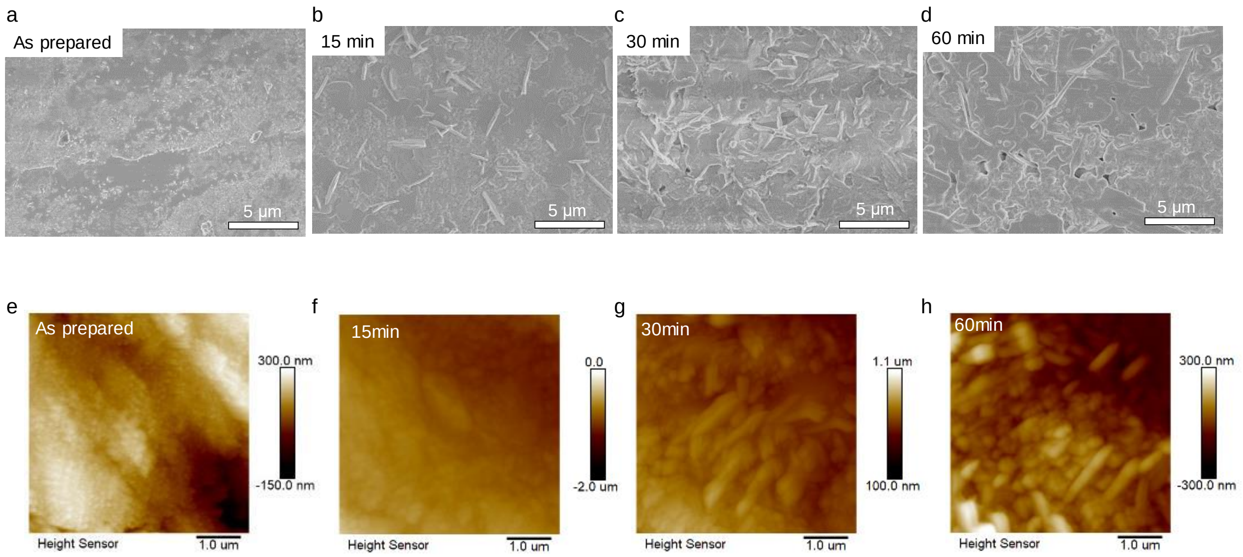

Supplementary Figure 14: Morphologies of the tablets with heat time increasing. a-d, SEM characterization of the morphology variation with heat-treat time. **e-h,** AFM measurements of the morphology variation with heat-treat time.

To confirm the thermal stability of the A-1D tablets, T-2D tablets, and F-1D tablets, Thermogravimetric analysis (TGA) measurements were performed, and the full spectra are shown in Supplementary Figure 15a. Supplementary Figure 15b is the amplified spectra of the selected part in Supplementary Figure 15a. The initial decomposition temperatures of all the TA-based perovskite tablets are higher than 200℃, while the TAI tablet is only 131℃, representing the strong bonding between TAI and PbI_2.

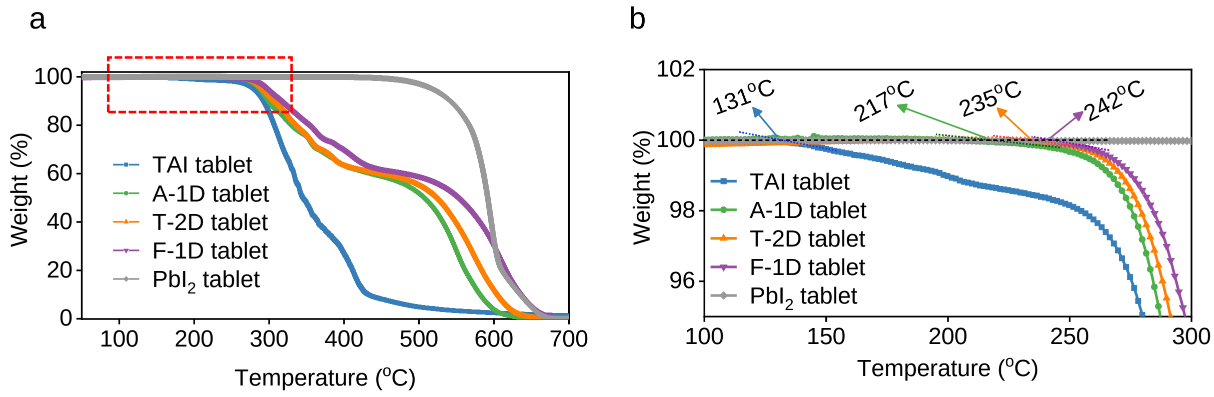

Supplementary Figure 15: TGA measurements. a, the complete TGA spectra of TAI, PbI_2, A-1D, T-2D, and F-1D tablets. **b,** the amplified spectra of the selected part with red dash line in Supplementary Figure 15a.

The indentation curves of the tablets are shown in Supplementary Figure 16, and we can see that all the indentation depths of $MAPbI_3$, PEA_2PbI_4, and $Cs_2AgBiBr_6$ perovskites are deeper than 3000 nm. In contrast, TA-based perovskite tablets show shallower than 3000 nm with more robust resistance to plastic deformation.

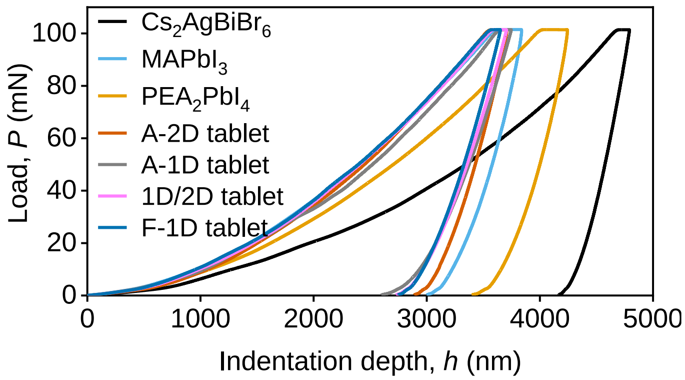

Supplementary Figure 16: Nanoindentation measurements. Nanoindentation curves of classical 3D $MAPbI_3$, 2D PEA_2PbI_4, all-inorganic $Cs_2AgBiBr_6$, and TA-based perovskite tablets.

The F-1D tablets were obtained after various water-soaking times, and then we dried the tablets on a hot plate at the temperature of 120℃ for ~3 hours. After this, the dried tablets were fabricated into the device with the structure of Au/perovskite tablet/C_{60}/BCP/Cr as the active layers. The corresponding response sensitivity to hard X-ray with a tube voltage of 120 kV_p is plotted in Figure 4b, and the sensitivity fitting lines are shown in Supplementary Figure 17.

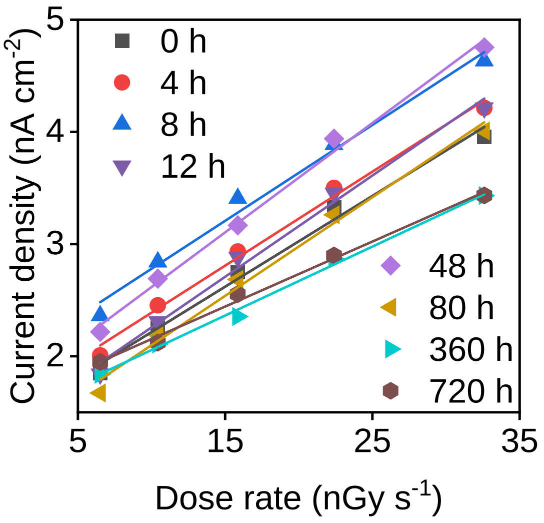

Supplementary Figure 17: Sensitivity fitting lines of the tablets after various soaking hours in water.

In order to explore the long-term soaking stability of the tablet material in water, we soaked a tablet with 2D structure in water for 48 days. After soaking for only 1 minute, the 2D tablet started to transfer to the 1D structure and kept the 1D structure for the next 48 days.

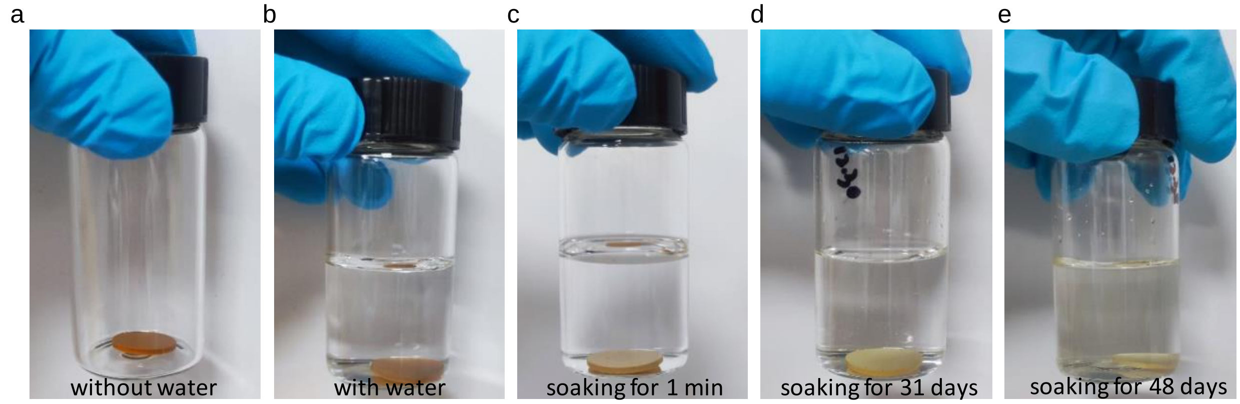

Supplementary Figure 18: Long-term water stability of the 2D tablet. a, image of the as-prepared 2D tablet. **b-c,** images of the 2D tablet after soaking into the water for 0 and 1 minute. **d-e,** images of the 2D tablet after soaking into the water for 31 and 48 days.

NED characterization. To confirm the minimization dose to the medical imaging patient, we performed NED value of the F-1D flat panel detector based on the reported noise experiments method[4], the resulted NED of 118 nGy is comparable to the reported scintillators[5].

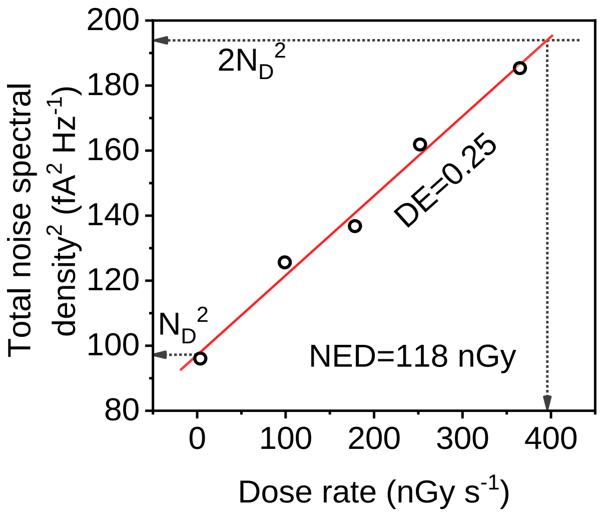

Supplementary Figure 19: NED of the F-1D flat panel detector. The integration time is 300 ms.

The sensitivity of A-1D (Supplementary Figure 20a) and F-1D tablets (Supplementary Figure 20b) to the hard X-ray of 120 kV_p was characterized under different operating temperatures. Comparing the two figures, we can see the larger response current in the F-1D case than in the A-

1D case. And when the operating temperature was up to 150℃, an unstable baseline occurred in the A-1D case, while the F-1D remained stable. The F-1D tablets are more thermally stable.

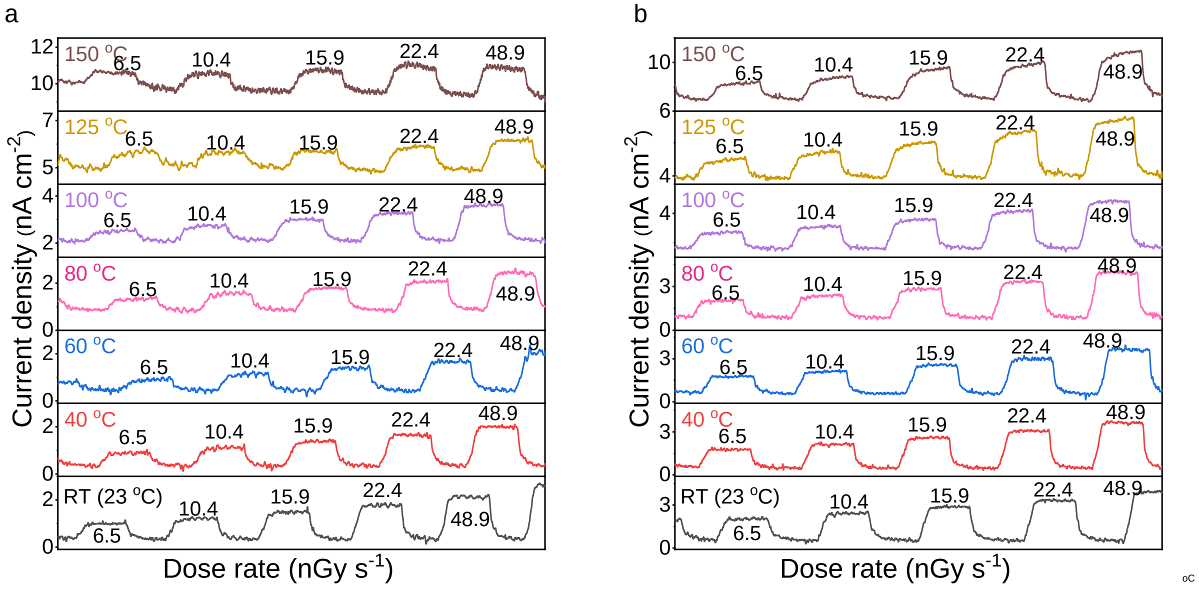

Supplementary Figure 20: Temperature-dependent X-ray response characterization. On-off response of A-1D tablet (**a**) and F-1D table (**b**) to hard X-ray with tube voltage of 120 kV_p.

Based on the on-off raw data shown in Supplementary Figure 20, we obtained the signal current by subtracting the adjacent dark current (off) from the radiated current (on) and plotted the signal current under different radiation dose rates in Supplementary Figure 21. Then the signal plots were fitted in a line, and the slope represented the sensitivity of the tablet material. The corresponding sensitivities under different temperatures were plotted in Figure 4d, which showed

the increased tendency of sensitivity with increasing temperature in the F-1D case. In contrast, a decreased tendency occurred in the A-1D case.

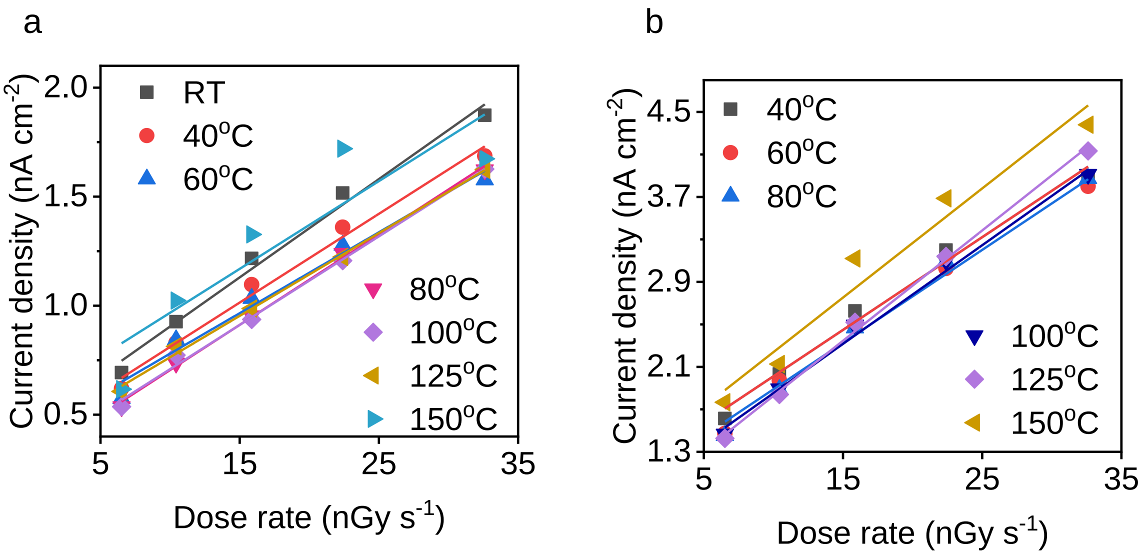

Supplementary Figure 21: Temperature-dependent X-ray response sensitivity. Sensitivity fitting of the A-1D (**a**) and F-1D tablets (**b**) under various operating temperatures.

μτ product, which determines the charge collection capability and device signal intensity, is an essential parameter for an X-ray detector. We derived the μτ product by fitting the X-ray photoconductivity curves to the modified Hecht equation[6, 7]. The corresponding fitting curves are shown in Supplementary Figure 22. The A-1D tablet had a μτ product of 1.0×10^{-4} cm^2 V^{-1}, while the F-1D tablet had a larger μτ product of 1.8×10^{-4} cm^2 V^{-1}, which can be explained by the relaxed microstrain and pinhole-free characteristics of the F-1D tablet.

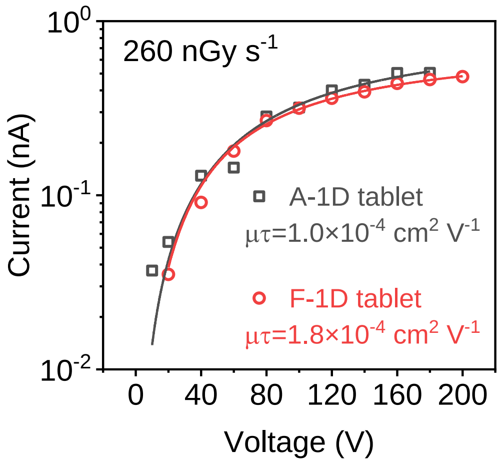

Supplementary Figure 22: μτ product fitting of A-1D and F-1D tablet.

The robust crystal alignments within the F-1D tablet enable the X-ray detector to have a high resistance to ion migration, even in a high electric field with an intensity of 800 V mm^{-1} (bias voltage: 1000 V). And the detector performs a much higher sensitivity than the working condition of 160 V mm^{-1} (bias voltage: 200 V), as shown in Supplementary Figure 23.

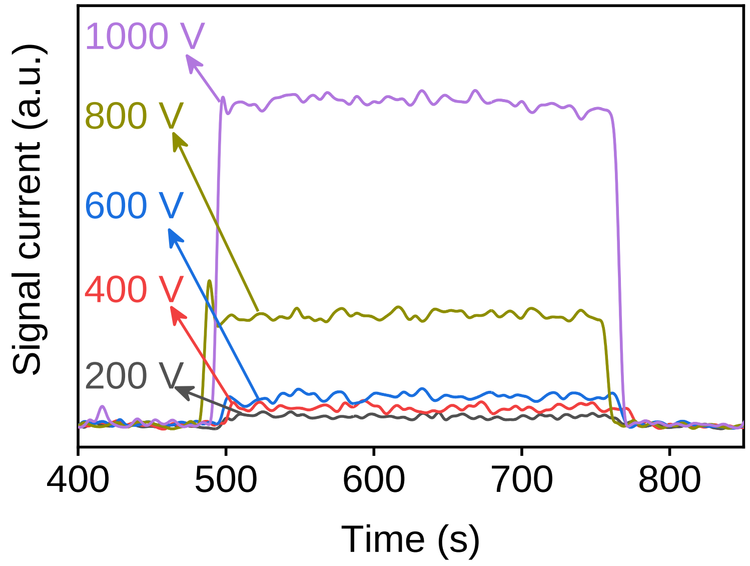

Supplementary Figure 23: Signal current curve with bias voltage.

SCLC measurements. To confirm the trapped carrier type and transport carrier type in the F-1D detector, we performed SCLC measurements. The electron-only device is fabricated with the device structure of Cr/C_{60}/perovskite/C_{60}/Cr (Supplementary Figure 24a) while the hole-only device is fabricated with the device structure of Au/MoO_3/perovskite/MoO_3/Au (Supplementary Figure 24b). The thickness of C_{60} and MoO_3 is 25 nm, and the thickness of electrodes Au and Cr is 30 nm. The hole-only device has two orders of magnitude larger current than that of electron-only device (Supplementary Figure 24c), representing that holes is transport carriers and electron is the trapped carrier.

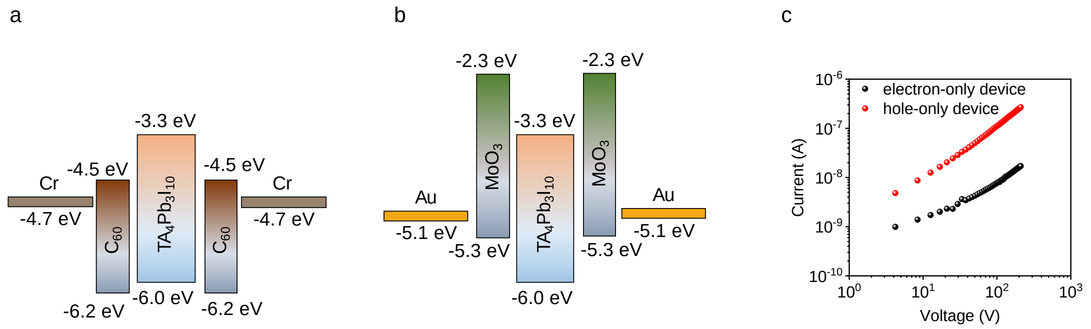

Supplementary Figure 24: SCLC measurements. a-b, diagrams of the electron-only and hole-only device. **c,** SCLC characterization of the F-1D detector.

Respond speed. The response time of the detector is characterized to evaluate the detector response speed, and response time of τ_{on} of 172 μs and τ_{off} of 128 μs is derived from the on/off response of an oscilloscope for the detector. The gain factor (*G*) can be described by:

$$G=\frac{\tau}{t} \quad (1)$$

where τ is the charge carrier lifetime, which determines the device response time. *t* is the charge carrier transit time, which can be calculated by:

$$t=\frac{d^2}{\mu\times V}=\frac{d}{\mu\times E} \quad (2)$$

Therefore, a large charge carrier mobility and electric field product can result in a small transit time and a large device gain, consistent with the 1D $TA_4Pb_3I_{10}$ perovskite performance. It should be noted that a large device gain doesn't require a very long charge carrier lifetime, since shallow traps can also lead to a large gain and fast response speed[8]

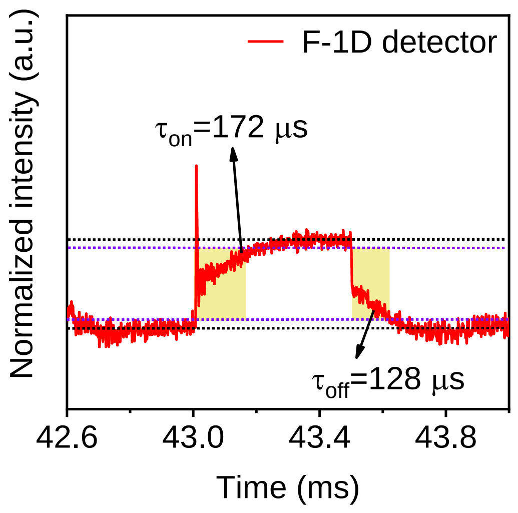

Supplementary Figure 25: Response speed measurement of the F-1D detector.

Anisotropic property. As the low-dimensional materials have anisotropic carrier transport properties, we confirmed the anisotropic carrier transport of $TA_4Pb_3I_{10}$ single crystal and F-1D tablet. Supplementary Figure 26a gives the different planes of the $TA_4Pb_3I_{10}$ single crystal, Supplementary Figure 26b depicts the response signals of three carrier transport directions to X-ray with the tube voltage of 120 kV_p and the dose rate of 1.01 μC Gy^{-1} cm^{-2}. The area of the single crystal device is 1 mm^2, the thickness of the single crystal is 1.2 mm, and the applied bias voltage is 200 V. Supplementary Figure 26c shows the diagram of tablet and Supplementary Figure 26d exhibits the anisotropic properties of F-1D tablet, a larger signal can be achieved in the direction of in plane than the direction of out of plane. The area of the tablet device is 1 mm^2, the thickness of the tablet is 0.4 mm, and the applied bias voltage is 200 V. Finally, we conclude the sensitivity variation with applied electric field intensity (Supplementary Figure 26e).

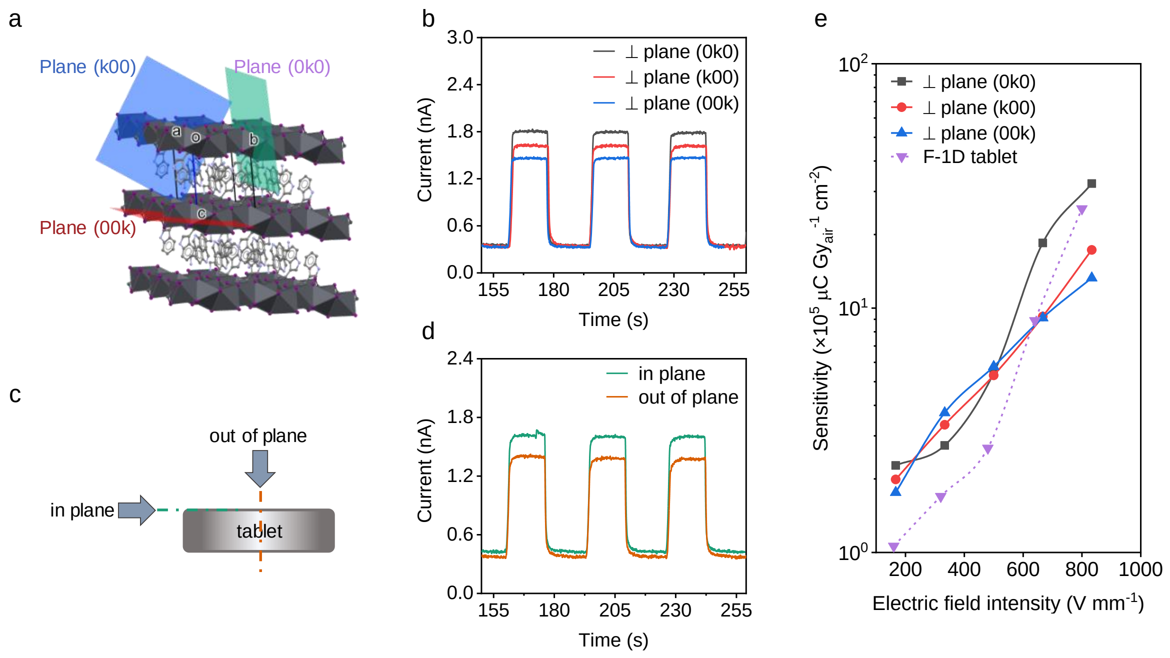

Supplementary Figure 26: Anisotropic measurements. Anisotropic measurements of the $TA_4Pb_3I_{10}$ single crystal (**a, b**) and tablet (**c, d**). Summary of the sensitivity variation with applied electric field intensity (**e**), and the tablet data here is figure 4f in manuscript.

In our imaging system, to conveniently connect the electrode arrays on the X-ray imager with the computer, we design a probe card as the signal readout terminal, as shown in Supplementary Figure 27.

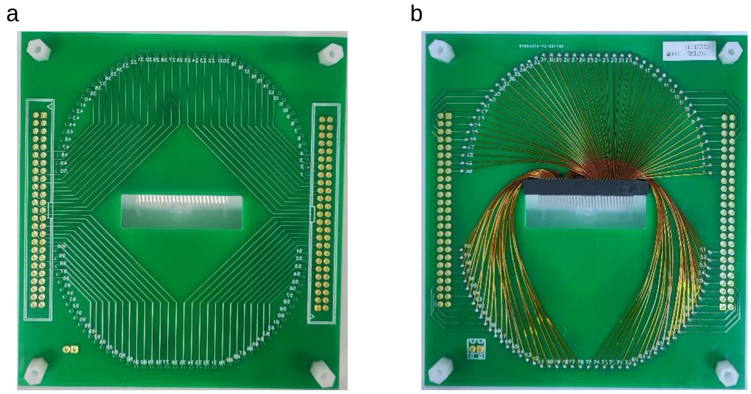

Supplementary Figure 27: Photos of the Signal readout probe card. a, the front side of the probe card. **b,** the back side of the probe card, where probe body arranged.

SUPPLEMENTARY TABLE

We summarized the Anisotropic Displacement Parameters ($\bar{\mathbf{U}}_{\mathbf{eq}}$) of $MAPbI_3$, PEA_2PbI_4, $Cs_2AgBiBr_6$, and $TA_4Pb_3I_{10}$ in Supplementary Table 1. The Anisotropic Displacement Parameters were derived from single-crystal diffraction experiments. $MAPbI_3$, PEA_2PbI_4, and $Cs_2AgBiBr_6$ were referred to in the previous reports[1-3].

Supplementary Table 1. Anisotropic Displacement Parameters ($\bar{\mathbf{U}}_{\mathbf{eq}}$, Å^2×10^3) for single crystals.

$\bar{\mathbf{U}}_{\mathbf{eq}}$ (Å^2×10^3)	Pb/Bi	I/Br	N/Cs	C/Ag
$MAPbI_3$	32.0	81.0	170.0	340.0
PEA_2PbI_4	19.8	25.9	26.4	24.7

$Cs_2AgBiBr_6$	17.9	26.4	49.7	18.0
$TA_4Pb_3I_{10}$	14.0	16.7	17.2	17.6

SUPPLEMENTARY METHODS

Materials. All the commercial materials were used as received without further purification, including Tryptamine ($C_{10}N_2H_{12}$, expressed as TA, 98%, Energy Chemical), Hydroiodic acid (HI, 57 wt% in water, Energy Chemical), PbI_2 (99%, Xi'an Polymer Light Technology Corp), Sodium hydroxide (Sinopharm Chemical Reagent Co., Ltd) Acetonitrile (C_2H_3N, ACN, AR Beijing Chemical Works), Dichloromethane (DCM, AR Beijing Chemical Works), Ethyl acetate (EA, AR Beijing Chemical Works). Positive photoresist (aqueous solution) was purchased from Kempur Microelectronics Inc. Gold etchant (liquid) was purchased from Alfa Asear.

Synthesis of tryptamine hydroiodide: $C_{10}N_2H_{12}$·HI. TA (10 g, 62.5 mmol) was dissolved in ~50 mL EtOH and stirred under an ice bath. HI aqueous solution (8.5 mL, 62.5 mmol) was dropwise into the solution, and stirred for ~5 h. The reaction solution was condensed under reduced pressure and dissolved in a small amount of EtOH. Recrystallized the $C_{10}N_2H_{12}$·HI (abbreviated as TAI) from EtOH with DCM as an inferior solvent and repeated this purification 3-4 times, white granular product can be obtained (8.13 g, yield 45%). ^{1}H NMR (400 MHz, DMSO) δ 10.98 (s, 1H), 7.74 (s, 2H), 7.57 (d, J = 7.8 Hz, 1H), 7.39 (d, J = 8.1 Hz, 1H), 7.26 (d, J = 1.9 Hz, 1H), 7.12 (t, J = 7.5 Hz, 1H), 7.03 (t, J = 7.4 Hz, 1H), 3.17 – 3.05 (m, 2H), 3.04 – 2.95 (m, 2H). ^{13}C NMR (101 MHz, DMSO) δ 136.67, 127.25, 123.96, 121.80, 119.11, 118.69, 112.05, 109.71, 23.52.

Growth of 1D $(C_{10}N_2H_{15})_4Pb_3I_{10}$ crystal. TAI (1.722 g, 6 mmol) and PbI_2 (1.383 g, 3 mmol) were dissolved in 3 mL ACN, and sonicated for ~0.5 h under room temperature to make a clear solution.

Then the solution was filtered in a 20 mL glass bottle with a 0.22 μm filter immediately. The bottle was sealed and put on a heat plate of 60°C. After ~2 h, the crystals with yellowish-brown or light-yellow color begin to deposit (The specific precipitation color is related to precipitation temperature, solution concentration, and raw material purity, but their XRD spectra are consistent).

Growth of 2D $(C_{10}N_2H_{15})_2PbI_4$ crystal. 1) TAI (1.722 g, 6 mmol) and PbI_2 (1.383 g, 3 mmol) were dissolved in 3 mL ACN, and sonicated for ~0.5 h under room temperature to make a clear solution. Then the solution was filtered in a 20 mL glass bottle with a 0.22 μm filter immediately. The solution was sealed in a bottle and put on a heat plate of 70°C. After ~15 minutes, the crystals with organ color begin to deposit; 2) TAI (1.722 g, 6 mmol) and PbI_2 (1.383 g, 3 mmol) were dissolved in 3 mL ACN, and sonicated for ~0.5 h under room temperature to make a clear solution. Then the solution was filtered in a 20 mL glass bottle with a 0.22 μm filter immediately. Add 3 mL EA to the filtered solution in a sealed bottle, then put the solution on a heat plate of 70°C. After ~15 minutes, the crystals begin to deposit; 3) TAI (1.722 g, 6 mmol) and PbI_2 (1.383 g, 3 mmol) were dissolved in 3 mL ACN, and sonicated for ~0.5 h under room temperature to make a clear solution. Then the solution was filtered in a 20 mL glass bottle with a 0.22 μm filter immediately, and put in EA bath for ~24 h, the crystals begin to deposit.

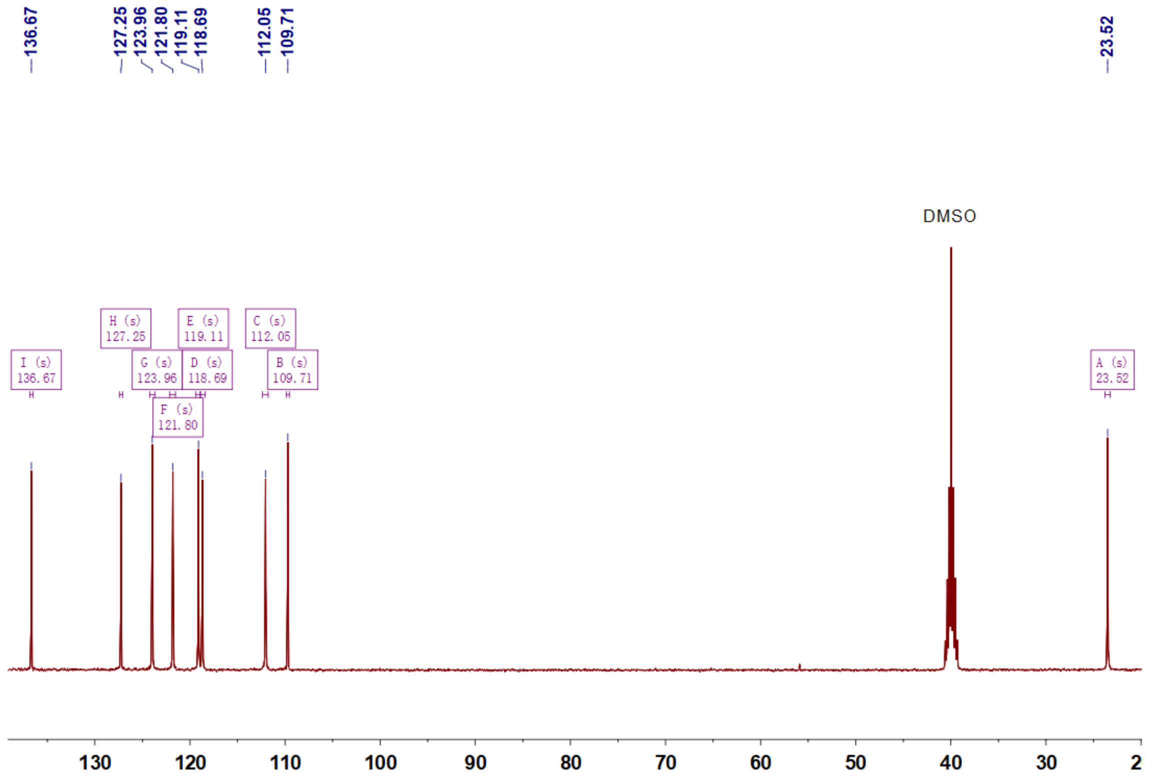

Supplementary Figure 28: ^{13}C NMR spectrum of TAI in DMSO.

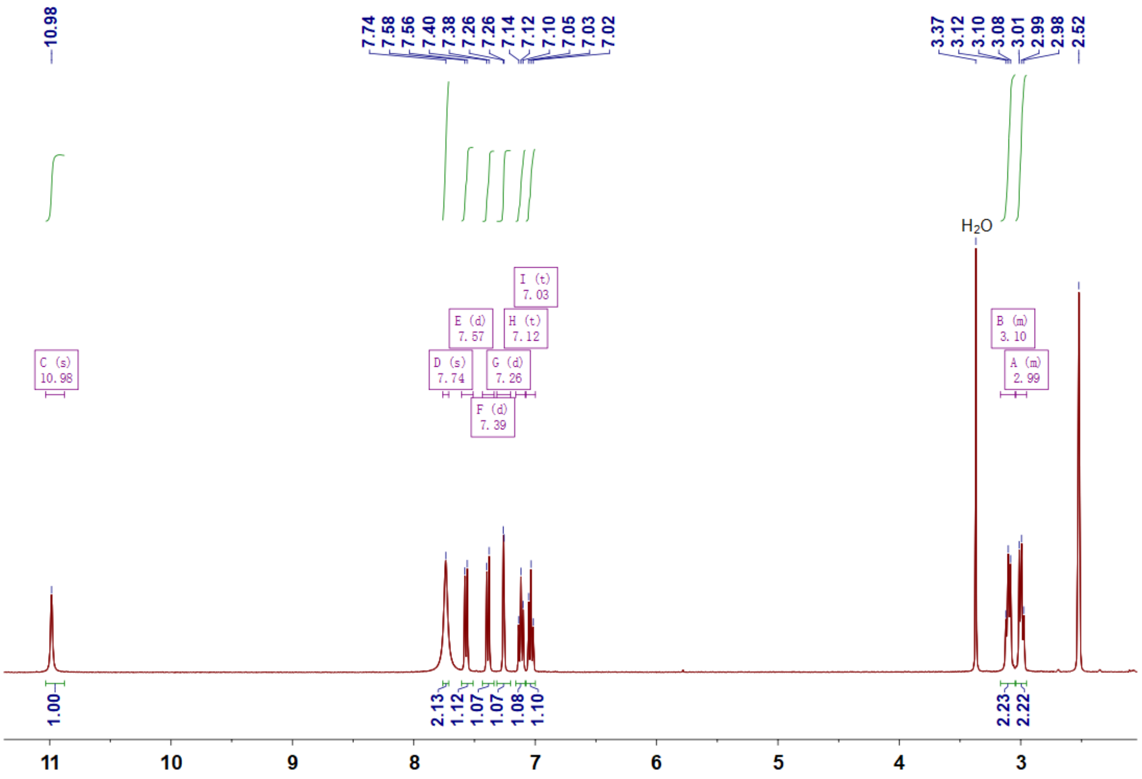

Supplementary Figure 29: ^{1}H NMR spectrum of TAI in DMSO.

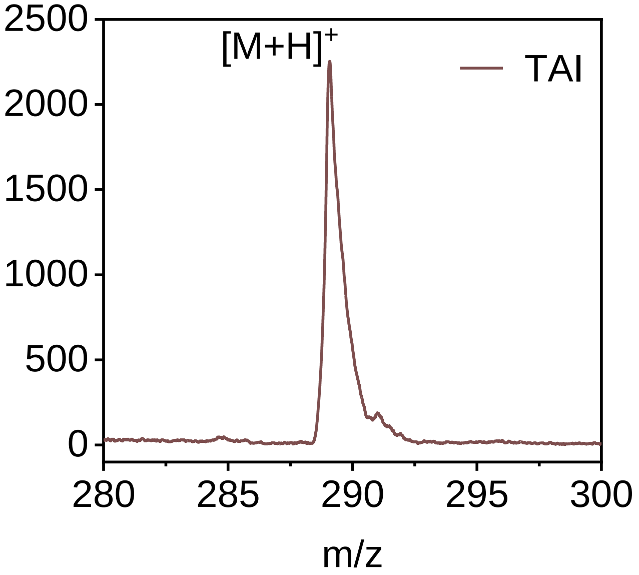

Supplementary Figure 30: MALDI-TOF mass spectra of TAI.

Supplementary References

1. Stoumpos, C.C., Malliakas, C.D. & Kanatzidis, M.G. Semiconducting Tin and Lead Iodide Perovskites with Organic Cations: Phase Transitions, High Mobilities, and Near-Infrared Photoluminescent Properties. *Inorganic Chemistry* **52**, 9019-9038 (2013).
2. Slavney, A.H., Hu, T., Lindenberg, A.M. & Karunadasa, H.I. A Bismuth-Halide Double Perovskite with Long Carrier Recombination Lifetime for Photovoltaic Applications. *Journal of the American Chemical Society* **138**, 2138-2141 (2016).
3. Du, K.-z. et al. Two-Dimensional Lead(II) Halide-Based Hybrid Perovskites Templated by Acene Alkylamines: Crystal Structures, Optical Properties, and Piezoelectricity. *Inorganic Chemistry* **56**, 9291-9302 (2017).
4. Sakhatskyi, K. et al. Stable perovskite single-crystal X-ray imaging detectors with single-photon sensitivity. *Nature Photonics* **17**, 510-517 (2023).
5. Job, I., Boyce, S., Petrillo, M. & Zhou, K. A comparison of quantum limited dose and noise equivalent dose. (2016).
6. Androulakis, J. et al. Dimensional Reduction: A Design Tool for New Radiation Detection Materials. *Advanced Materials* **23**, 4163-4167 (2011).
7. Wei, H. et al. Sensitive X-ray detectors made of methylammonium lead tribromide perovskite single crystals. *Nature Photonics* **10**, 333-339 (2016).
8. Wei, H., Fang, Y., Yuan, Y., Shen, L. & Huang, J. Trap Engineering of CdTe Nanoparticle for High Gain, Fast Response, and Low Noise P3HT:CdTe Nanocomposite Photodetectors. *Advanced Materials* **27**, 4975-4981 (2015).
