# Supplementary material for: Cation-π interactions enabled water-stable perovskite X-ray flat mini-panel imager
Source: Nat Commun. 2024 Jan 4;15:257. doi: 10.1038/s41467-023-44644-7 (PMC10767000; doi:10.1038/s41467-023-44644-7)
Supplement: Supplementary file 3 — Description of Additional Supplementary Files [file 41467_2023_44644_MOESM3_ESM.pdf]

Description of Additional Supplementary Files

Supplementary Movie 1 The dimension transition process of TA-based perovskite in water. And water soaking stability comparison between BA_2PbI_4, PEA_2PbI_4 and TA-based perovskite.

Supplementary Movie 2 The photolithography process of a rectangular perovskite tablet with size of 2.4*3.6 cm.

Supplementary Data 1 The atomic coordinates of the optimized computational models and molecular dynamics trajectories at the initial and final configurations.
