# Supplementary material for: Cation-π interactions enabled water-stable perovskite X-ray flat mini-panel imager
Source: Nat Commun. 2024 Jan 4;15:257. doi: 10.1038/s41467-023-44644-7 (PMC10767000; doi:10.1038/s41467-023-44644-7)
Supplement: Supplementary file 6 — Supplementary Data 1 [file 41467_2023_44644_MOESM6_ESM.zip › DFT simulation/molecular dynamic trajectories.docx]

Molecular dynamics trajectories of 1D at the initial configurations.

#======================================================================

# CRYSTAL DATA

#----------------------------------------------------------------------

data_VESTA_phase_1

_chemical_name_common 'I10 Pb3, 4(C10 H13 N2)'

_cell_length_a 9.483500

_cell_length_b 11.834500

_cell_length_c 13.711300

_cell_angle_alpha 75.258003

_cell_angle_beta 83.017998

_cell_angle_gamma 78.936996

_cell_volume 1456.246579

_space_group_name_H-M_alt 'P 1'

_space_group_IT_number 1

loop_

_space_group_symop_operation_xyz

'x, y, z'

loop_

_atom_site_label

_atom_site_occupancy

_atom_site_fract_x

_atom_site_fract_y

_atom_site_fract_z

_atom_site_adp_type

_atom_site_B_iso_or_equiv

_atom_site_type_symbol

Pb1 1.0 0.000000 0.000000 0.000000 Biso 1.000000 Pb

Pb2 1.0 0.094540 0.662940 0.958830 Biso 1.000000 Pb

Pb3 1.0 0.905460 0.337060 0.041170 Biso 1.000000 Pb

I1 1.0 0.099790 0.439330 0.153770 Biso 1.000000 I

I2 1.0 0.900210 0.560670 0.846230 Biso 1.000000 I

I3 1.0 0.374700 0.537770 0.854320 Biso 1.000000 I

I4 1.0 0.625300 0.462230 0.145680 Biso 1.000000 I

I5 1.0 0.806970 0.792390 0.077530 Biso 1.000000 I

I6 1.0 0.193030 0.207610 0.922470 Biso 1.000000 I

I7 1.0 0.264490 0.798360 0.087230 Biso 1.000000 I

I8 1.0 0.735510 0.201640 0.912770 Biso 1.000000 I

I9 1.0 0.957290 0.092990 0.202870 Biso 1.000000 I

I10 1.0 0.042710 0.907010 0.797130 Biso 1.000000 I

C1 1.0 0.511500 0.880100 0.443900 Biso 1.000000 C

C2 1.0 0.488500 0.119900 0.556100 Biso 1.000000 C

C3 1.0 0.468300 0.831600 0.544300 Biso 1.000000 C

C4 1.0 0.531700 0.168400 0.455700 Biso 1.000000 C

C5 1.0 0.412600 0.950200 0.373800 Biso 1.000000 C

C6 1.0 0.587400 0.049800 0.626200 Biso 1.000000 C

C7 1.0 0.267200 0.975500 0.407500 Biso 1.000000 C

C8 1.0 0.732800 0.024500 0.592500 Biso 1.000000 C

C9 1.0 0.495500 0.979900 0.277900 Biso 1.000000 C

C10 1.0 0.504500 0.020100 0.722100 Biso 1.000000 C

C11 1.0 0.444100 0.060900 0.180200 Biso 1.000000 C

C12 1.0 0.555900 0.939100 0.819800 Biso 1.000000 C

C13 1.0 0.635500 0.929200 0.294300 Biso 1.000000 C

C14 1.0 0.364500 0.070800 0.705700 Biso 1.000000 C

C15 1.0 0.225800 0.929100 0.507700 Biso 1.000000 C

C16 1.0 0.774200 0.070900 0.492300 Biso 1.000000 C

C17 1.0 0.324600 0.857600 0.575100 Biso 1.000000 C

C18 1.0 0.675400 0.142400 0.424900 Biso 1.000000 C

C19 1.0 0.454300 0.187500 0.179600 Biso 1.000000 C

C20 1.0 0.545700 0.812500 0.820400 Biso 1.000000 C

C21 1.0 0.820700 0.587300 0.492600 Biso 1.000000 C

C22 1.0 0.179300 0.412700 0.507400 Biso 1.000000 C

C23 1.0 0.860500 0.655100 0.551300 Biso 1.000000 C

C24 1.0 0.139500 0.344900 0.448700 Biso 1.000000 C

C25 1.0 0.961500 0.688200 0.344400 Biso 1.000000 C

C26 1.0 0.038500 0.311800 0.655600 Biso 1.000000 C

C27 1.0 0.998800 0.753600 0.404300 Biso 1.000000 C

C28 1.0 0.001200 0.246400 0.595700 Biso 1.000000 C

C29 1.0 0.872000 0.605600 0.389000 Biso 1.000000 C

C30 1.0 0.128000 0.394400 0.611000 Biso 1.000000 C

C31 1.0 0.949500 0.736400 0.507300 Biso 1.000000 C

C32 1.0 0.050500 0.263600 0.492700 Biso 1.000000 C

C33 1.0 0.737400 0.460800 0.425300 Biso 1.000000 C

C34 1.0 0.262600 0.539200 0.574700 Biso 1.000000 C

C35 1.0 0.734300 0.495500 0.513400 Biso 1.000000 C

C36 1.0 0.265700 0.504500 0.486600 Biso 1.000000 C

C37 1.0 0.656000 0.450200 0.614200 Biso 1.000000 C

C38 1.0 0.344000 0.549800 0.385800 Biso 1.000000 C

C39 1.0 0.589800 0.343300 0.614600 Biso 1.000000 C

C40 1.0 0.410200 0.656700 0.385400 Biso 1.000000 C

H1 1.0 0.535554 0.782471 0.589780 Biso 1.000000 H

H2 1.0 0.464446 0.217529 0.410220 Biso 1.000000 H

H3 1.0 0.198593 0.023366 0.362609 Biso 1.000000 H

H4 1.0 0.801407 0.976634 0.637391 Biso 1.000000 H

H5 1.0 0.725151 0.826007 0.419408 Biso 1.000000 H

H6 1.0 0.274849 0.173993 0.580592 Biso 1.000000 H

H7 1.0 0.343144 0.054745 0.173982 Biso 1.000000 H

H8 1.0 0.656856 0.945255 0.826018 Biso 1.000000 H

H9 1.0 0.504377 0.037311 0.122155 Biso 1.000000 H

H10 1.0 0.495623 0.962689 0.877845 Biso 1.000000 H

H11 1.0 0.714220 0.935938 0.244422 Biso 1.000000 H

H12 1.0 0.285780 0.064062 0.755578 Biso 1.000000 H

H13 1.0 0.127619 0.946364 0.531485 Biso 1.000000 H

H14 1.0 0.872381 0.053636 0.468515 Biso 1.000000 H

H15 1.0 0.292011 0.826360 0.643267 Biso 1.000000 H

H16 1.0 0.707989 0.173640 0.356733 Biso 1.000000 H

H17 1.0 0.383145 0.214142 0.231647 Biso 1.000000 H

H18 1.0 0.616855 0.785858 0.768353 Biso 1.000000 H

H19 1.0 0.551509 0.190093 0.197426 Biso 1.000000 H

H20 1.0 0.448491 0.809907 0.802574 Biso 1.000000 H

H21 1.0 0.826590 0.645318 0.620545 Biso 1.000000 H

H22 1.0 0.173410 0.354682 0.379455 Biso 1.000000 H

H23 1.0 0.995670 0.699087 0.275119 Biso 1.000000 H

H24 1.0 0.004330 0.300913 0.724881 Biso 1.000000 H

H25 1.0 0.058678 0.811395 0.375671 Biso 1.000000 H

H26 1.0 0.941322 0.188605 0.624329 Biso 1.000000 H

H27 1.0 0.978333 0.781693 0.547146 Biso 1.000000 H

H28 1.0 0.021667 0.218307 0.452854 Biso 1.000000 H

H29 1.0 0.829991 0.522865 0.286178 Biso 1.000000 H

H30 1.0 0.170009 0.477135 0.713822 Biso 1.000000 H

H31 1.0 0.691620 0.399314 0.417494 Biso 1.000000 H

H32 1.0 0.308380 0.600686 0.582506 Biso 1.000000 H

H33 1.0 0.579052 0.513999 0.629708 Biso 1.000000 H

H34 1.0 0.420948 0.486001 0.370292 Biso 1.000000 H

H35 1.0 0.724248 0.428433 0.667727 Biso 1.000000 H

H36 1.0 0.275752 0.571567 0.332273 Biso 1.000000 H

H37 1.0 0.494287 0.248258 0.030853 Biso 1.000000 H

H38 1.0 0.505713 0.751742 0.969147 Biso 1.000000 H

H39 1.0 0.432343 0.345675 0.081637 Biso 1.000000 H

H40 1.0 0.567657 0.654325 0.918363 Biso 1.000000 H

H41 1.0 0.337603 0.268739 0.061882 Biso 1.000000 H

H42 1.0 0.662397 0.731261 0.938118 Biso 1.000000 H

H43 1.0 0.464610 0.240041 0.710189 Biso 1.000000 H

H44 1.0 0.535390 0.759959 0.289811 Biso 1.000000 H

H45 1.0 0.423740 0.365910 0.716131 Biso 1.000000 H

H46 1.0 0.576260 0.634090 0.283869 Biso 1.000000 H

H47 1.0 0.553207 0.291456 0.764449 Biso 1.000000 H

H48 1.0 0.446793 0.708544 0.235551 Biso 1.000000 H

H49 1.0 0.529728 0.362388 0.556492 Biso 1.000000 H

H50 1.0 0.470272 0.637612 0.443508 Biso 1.000000 H

H51 1.0 0.667543 0.276575 0.606566 Biso 1.000000 H

H52 1.0 0.332457 0.723425 0.393434 Biso 1.000000 H

N1 1.0 0.645600 0.867300 0.392900 Biso 1.000000 N

N2 1.0 0.354400 0.132700 0.607100 Biso 1.000000 N

N3 1.0 0.816700 0.528200 0.349600 Biso 1.000000 N

N4 1.0 0.183300 0.471800 0.650400 Biso 1.000000 N

N5 1.0 0.426900 0.270700 0.078550 Biso 1.000000 N

N6 1.0 0.573100 0.729300 0.921450 Biso 1.000000 N

N7 1.0 0.498900 0.306600 0.710800 Biso 1.000000 N

N8 1.0 0.501100 0.693400 0.289200 Biso 1.000000 N

Molecular dynamics trajectories of 1D at the final configurations.

#======================================================================

# CRYSTAL DATA

#----------------------------------------------------------------------

data_VESTA_phase_1

_chemical_name_common 'I10 Pb3, 4(C10 H13 N2) '

_cell_length_a 9.780734

_cell_length_b 11.963501

_cell_length_c 13.870609

_cell_angle_alpha 74.886246

_cell_angle_beta 82.948586

_cell_angle_gamma 78.656006

_cell_volume 1531.888202

_space_group_name_H-M_alt 'P 1'

_space_group_IT_number 1

loop_

_space_group_symop_operation_xyz

'x, y, z'

loop_

_atom_site_label

_atom_site_occupancy

_atom_site_fract_x

_atom_site_fract_y

_atom_site_fract_z

_atom_site_adp_type

_atom_site_B_iso_or_equiv

_atom_site_type_symbol

Pb1 1.0 0.000000 0.000000 0.000000 Biso 1.000000 Pb

Pb2 1.0 0.096136 0.661230 0.957535 Biso 1.000000 Pb

Pb3 1.0 0.903863 0.338770 0.042465 Biso 1.000000 Pb

I1 1.0 0.097201 0.438311 0.153942 Biso 1.000000 I

I2 1.0 0.902799 0.561689 0.846058 Biso 1.000000 I

I3 1.0 0.378867 0.535364 0.856682 Biso 1.000000 I

I4 1.0 0.621133 0.464636 0.143318 Biso 1.000000 I

I5 1.0 0.813156 0.791242 0.070808 Biso 1.000000 I

I6 1.0 0.186844 0.208758 0.929192 Biso 1.000000 I

I7 1.0 0.264520 0.797170 0.081785 Biso 1.000000 I

I8 1.0 0.735480 0.202830 0.918215 Biso 1.000000 I

I9 1.0 0.951399 0.090554 0.203842 Biso 1.000000 I

I10 1.0 0.048601 0.909446 0.796158 Biso 1.000000 I

C1 1.0 0.509044 0.878533 0.442978 Biso 1.000000 C

C2 1.0 0.490956 0.121467 0.557022 Biso 1.000000 C

C3 1.0 0.466710 0.831214 0.543012 Biso 1.000000 C

C4 1.0 0.533290 0.168786 0.456988 Biso 1.000000 C

C5 1.0 0.412472 0.948093 0.372077 Biso 1.000000 C

C6 1.0 0.587528 0.051907 0.627923 Biso 1.000000 C

C7 1.0 0.270019 0.972627 0.404860 Biso 1.000000 C

C8 1.0 0.729981 0.027373 0.595140 Biso 1.000000 C

C9 1.0 0.492054 0.977623 0.277082 Biso 1.000000 C

C10 1.0 0.507946 0.022377 0.722918 Biso 1.000000 C

C11 1.0 0.443216 0.058858 0.180651 Biso 1.000000 C

C12 1.0 0.556784 0.941142 0.819349 Biso 1.000000 C

C13 1.0 0.629930 0.927103 0.294300 Biso 1.000000 C

C14 1.0 0.370070 0.072897 0.705700 Biso 1.000000 C

C15 1.0 0.228492 0.926608 0.504596 Biso 1.000000 C

C16 1.0 0.771508 0.073392 0.495404 Biso 1.000000 C

C17 1.0 0.325310 0.855865 0.572773 Biso 1.000000 C

C18 1.0 0.674690 0.144135 0.427227 Biso 1.000000 C

C19 1.0 0.452820 0.185570 0.179464 Biso 1.000000 C

C20 1.0 0.547180 0.814430 0.820536 Biso 1.000000 C

C21 1.0 0.815787 0.587898 0.492456 Biso 1.000000 C

C22 1.0 0.184213 0.412102 0.507544 Biso 1.000000 C

C23 1.0 0.853943 0.655654 0.550574 Biso 1.000000 C

C24 1.0 0.146057 0.344346 0.449426 Biso 1.000000 C

C25 1.0 0.953801 0.687248 0.344716 Biso 1.000000 C

C26 1.0 0.046199 0.312752 0.655284 Biso 1.000000 C

C27 1.0 0.989383 0.753213 0.404128 Biso 1.000000 C

C28 1.0 0.010617 0.246787 0.595872 Biso 1.000000 C

C29 1.0 0.867688 0.604362 0.389917 Biso 1.000000 C

C30 1.0 0.132312 0.395638 0.610083 Biso 1.000000 C

C31 1.0 0.940834 0.737198 0.506205 Biso 1.000000 C

C32 1.0 0.059166 0.262802 0.493795 Biso 1.000000 C

C33 1.0 0.737418 0.461190 0.425984 Biso 1.000000 C

C34 1.0 0.262582 0.538810 0.574016 Biso 1.000000 C

C35 1.0 0.732159 0.496709 0.513741 Biso 1.000000 C

C36 1.0 0.267841 0.503291 0.486259 Biso 1.000000 C

C37 1.0 0.658150 0.451351 0.613495 Biso 1.000000 C

C38 1.0 0.341850 0.548649 0.386505 Biso 1.000000 C

C39 1.0 0.593704 0.344543 0.616735 Biso 1.000000 C

C40 1.0 0.406296 0.655457 0.383265 Biso 1.000000 C

H1 1.0 0.542221 0.777412 0.595420 Biso 1.000000 H

H2 1.0 0.457779 0.222588 0.404580 Biso 1.000000 H

H3 1.0 0.193635 0.026538 0.352893 Biso 1.000000 H

H4 1.0 0.806365 0.973462 0.647107 Biso 1.000000 H

H5 1.0 0.730925 0.827620 0.424759 Biso 1.000000 H

H6 1.0 0.269075 0.172380 0.575241 Biso 1.000000 H

H7 1.0 0.335347 0.052844 0.170240 Biso 1.000000 H

H8 1.0 0.664653 0.947156 0.829760 Biso 1.000000 H

H9 1.0 0.509097 0.033472 0.116825 Biso 1.000000 H

H10 1.0 0.490903 0.966528 0.883175 Biso 1.000000 H

H11 1.0 0.723294 0.931655 0.243279 Biso 1.000000 H

H12 1.0 0.276706 0.068345 0.756721 Biso 1.000000 H

H13 1.0 0.119064 0.946029 0.531904 Biso 1.000000 H

H14 1.0 0.880936 0.053971 0.468096 Biso 1.000000 H

H15 1.0 0.288298 0.821566 0.650378 Biso 1.000000 H

H16 1.0 0.711702 0.178434 0.349622 Biso 1.000000 H

H17 1.0 0.374638 0.220101 0.233021 Biso 1.000000 H

H18 1.0 0.625362 0.779899 0.766979 Biso 1.000000 H

H19 1.0 0.556799 0.191095 0.197912 Biso 1.000000 H

H20 1.0 0.443201 0.808905 0.802088 Biso 1.000000 H

H21 1.0 0.819016 0.643168 0.629858 Biso 1.000000 H

H22 1.0 0.180984 0.356832 0.370142 Biso 1.000000 H

H23 1.0 0.990740 0.700197 0.265806 Biso 1.000000 H

H24 1.0 0.009260 0.299803 0.734194 Biso 1.000000 H

H25 1.0 0.055412 0.819192 0.371036 Biso 1.000000 H

H26 1.0 0.944588 0.180808 0.628963 Biso 1.000000 H

H27 1.0 0.972831 0.788865 0.551016 Biso 1.000000 H

H28 1.0 0.027169 0.211135 0.448984 Biso 1.000000 H

H29 1.0 0.848605 0.508986 0.283432 Biso 1.000000 H

H30 1.0 0.151395 0.491014 0.716568 Biso 1.000000 H

H31 1.0 0.694375 0.391231 0.410274 Biso 1.000000 H

H32 1.0 0.305625 0.608769 0.589726 Biso 1.000000 H

H33 1.0 0.576102 0.521538 0.633022 Biso 1.000000 H

H34 1.0 0.423898 0.478462 0.366978 Biso 1.000000 H

H35 1.0 0.732146 0.428591 0.672471 Biso 1.000000 H

H36 1.0 0.267854 0.571409 0.327529 Biso 1.000000 H

H37 1.0 0.508670 0.244791 0.025802 Biso 1.000000 H

H38 1.0 0.491330 0.755209 0.974198 Biso 1.000000 H

H39 1.0 0.438951 0.352865 0.081366 Biso 1.000000 H

H40 1.0 0.561049 0.647135 0.918634 Biso 1.000000 H

H41 1.0 0.334828 0.270156 0.053086 Biso 1.000000 H

H42 1.0 0.665172 0.729844 0.946914 Biso 1.000000 H

H43 1.0 0.474961 0.229076 0.719394 Biso 1.000000 H

H44 1.0 0.525039 0.770924 0.280606 Biso 1.000000 H

H45 1.0 0.431628 0.369729 0.729171 Biso 1.000000 H

H46 1.0 0.568372 0.630271 0.270829 Biso 1.000000 H

H47 1.0 0.578229 0.285153 0.774667 Biso 1.000000 H

H48 1.0 0.421771 0.714847 0.225333 Biso 1.000000 H

H49 1.0 0.519289 0.363378 0.559228 Biso 1.000000 H

H50 1.0 0.480711 0.636622 0.440772 Biso 1.000000 H

H51 1.0 0.673324 0.269129 0.606548 Biso 1.000000 H

H52 1.0 0.326676 0.730871 0.393452 Biso 1.000000 H

N1 1.0 0.639568 0.867474 0.392972 Biso 1.000000 N

N2 1.0 0.360432 0.132526 0.607028 Biso 1.000000 N

N3 1.0 0.816054 0.527421 0.351148 Biso 1.000000 N

N4 1.0 0.183946 0.472579 0.648852 Biso 1.000000 N

N5 1.0 0.430743 0.268332 0.078994 Biso 1.000000 N

N6 1.0 0.569257 0.731668 0.921006 Biso 1.000000 N

N7 1.0 0.513825 0.305186 0.715453 Biso 1.000000 N

N8 1.0 0.486175 0.694814 0.284547 Biso 1.000000 N

Molecular dynamics trajectories of 2D at the initial configurations.

#======================================================================

# CRYSTAL DATA

#----------------------------------------------------------------------

data_VESTA_phase_1

_chemical_name_common 'Autosave\of\3D\Atomistic\(8)'

_cell_length_a 18.500000

_cell_length_b 8.562000

_cell_length_c 8.727300

_cell_angle_alpha 90.000000

_cell_angle_beta 99.530998

_cell_angle_gamma 90.000000

_cell_volume 1363.296011

_space_group_name_H-M_alt 'P 1'

_space_group_IT_number 1

loop_

_space_group_symop_operation_xyz

'x, y, z'

loop_

_atom_site_label

_atom_site_occupancy

_atom_site_fract_x

_atom_site_fract_y

_atom_site_fract_z

_atom_site_adp_type

_atom_site_B_iso_or_equiv

_atom_site_type_symbol

I1 1.0 0.499450 0.686040 0.311980 Biso 1.000000 I

I2 1.0 0.500550 0.313960 0.688020 Biso 1.000000 I

I3 1.0 0.500550 0.186040 0.188020 Biso 1.000000 I

I4 1.0 0.499450 0.813960 0.811980 Biso 1.000000 I

I5 1.0 0.675920 0.014850 0.564710 Biso 1.000000 I

I6 1.0 0.324080 0.985150 0.435290 Biso 1.000000 I

I7 1.0 0.324080 0.514850 0.935290 Biso 1.000000 I

I8 1.0 0.675920 0.485150 0.064710 Biso 1.000000 I

C1 1.0 0.062970 0.524860 0.246850 Biso 1.000000 C

C2 1.0 0.937030 0.475140 0.753150 Biso 1.000000 C

C3 1.0 0.937030 0.024860 0.253150 Biso 1.000000 C

C4 1.0 0.062970 0.975140 0.746850 Biso 1.000000 C

C5 1.0 0.008850 0.445400 0.146400 Biso 1.000000 C

C6 1.0 0.991150 0.554600 0.853600 Biso 1.000000 C

C7 1.0 0.991150 0.945400 0.353600 Biso 1.000000 C

C8 1.0 0.008850 0.054600 0.646400 Biso 1.000000 C

C9 1.0 0.115110 0.446340 0.357520 Biso 1.000000 C

C10 1.0 0.884890 0.553660 0.642480 Biso 1.000000 C

C11 1.0 0.884890 0.946340 0.142480 Biso 1.000000 C

C12 1.0 0.115110 0.053660 0.857520 Biso 1.000000 C

C13 1.0 0.114020 0.282860 0.362070 Biso 1.000000 C

C14 1.0 0.885980 0.717140 0.637930 Biso 1.000000 C

C15 1.0 0.885980 0.782860 0.137930 Biso 1.000000 C

C16 1.0 0.114020 0.217140 0.862070 Biso 1.000000 C

C17 1.0 0.159580 0.566410 0.443300 Biso 1.000000 C

C18 1.0 0.840420 0.433590 0.556700 Biso 1.000000 C

C19 1.0 0.840420 0.066410 0.056700 Biso 1.000000 C

C20 1.0 0.159580 0.933590 0.943300 Biso 1.000000 C

C21 1.0 0.226620 0.545280 0.565950 Biso 1.000000 C

C22 1.0 0.773380 0.454720 0.434050 Biso 1.000000 C

C23 1.0 0.773380 0.045280 0.934050 Biso 1.000000 C

C24 1.0 0.226620 0.954720 0.065950 Biso 1.000000 C

C25 1.0 0.133610 0.706380 0.381580 Biso 1.000000 C

C26 1.0 0.866390 0.293620 0.618420 Biso 1.000000 C

C27 1.0 0.866390 0.206380 0.118420 Biso 1.000000 C

C28 1.0 0.133610 0.793620 0.881580 Biso 1.000000 C

C29 1.0 0.061120 0.204870 0.259820 Biso 1.000000 C

C30 1.0 0.938880 0.795130 0.740180 Biso 1.000000 C

C31 1.0 0.938880 0.704870 0.240180 Biso 1.000000 C

C32 1.0 0.061120 0.295130 0.759820 Biso 1.000000 C

C33 1.0 0.008980 0.284790 0.154050 Biso 1.000000 C

C34 1.0 0.991020 0.715210 0.845950 Biso 1.000000 C

C35 1.0 0.991020 0.784790 0.345950 Biso 1.000000 C

C36 1.0 0.008980 0.215210 0.654050 Biso 1.000000 C

C37 1.0 0.293720 0.531490 0.487640 Biso 1.000000 C

C38 1.0 0.706280 0.468510 0.512360 Biso 1.000000 C

C39 1.0 0.706280 0.031490 0.012360 Biso 1.000000 C

C40 1.0 0.293720 0.968510 0.987640 Biso 1.000000 C

H1 1.0 0.973210 0.500080 0.075260 Biso 1.000000 H

H2 1.0 0.026790 0.499920 0.924740 Biso 1.000000 H

H3 1.0 0.026790 0.000080 0.424740 Biso 1.000000 H

H4 1.0 0.973210 0.999920 0.575260 Biso 1.000000 H

H5 1.0 0.148780 0.226820 0.433720 Biso 1.000000 H

H6 1.0 0.851220 0.773180 0.566280 Biso 1.000000 H

H7 1.0 0.851220 0.726820 0.066280 Biso 1.000000 H

H8 1.0 0.148780 0.273180 0.933720 Biso 1.000000 H

H9 1.0 0.048920 0.756800 0.212800 Biso 1.000000 H

H10 1.0 0.951080 0.243200 0.787200 Biso 1.000000 H

H11 1.0 0.951080 0.256800 0.287200 Biso 1.000000 H

H12 1.0 0.048920 0.743200 0.712800 Biso 1.000000 H

H13 1.0 0.221370 0.449900 0.627170 Biso 1.000000 H

H14 1.0 0.778630 0.550100 0.372830 Biso 1.000000 H

H15 1.0 0.778630 0.949900 0.872830 Biso 1.000000 H

H16 1.0 0.221370 0.050100 0.127170 Biso 1.000000 H

H17 1.0 0.232050 0.635810 0.637430 Biso 1.000000 H

H18 1.0 0.767950 0.364190 0.362570 Biso 1.000000 H

H19 1.0 0.767950 0.135810 0.862570 Biso 1.000000 H

H20 1.0 0.232050 0.864190 0.137430 Biso 1.000000 H

H21 1.0 0.153470 0.805540 0.414260 Biso 1.000000 H

H22 1.0 0.846530 0.194460 0.585740 Biso 1.000000 H

H23 1.0 0.846530 0.305540 0.085740 Biso 1.000000 H

H24 1.0 0.153470 0.694460 0.914260 Biso 1.000000 H

H25 1.0 0.060210 0.093970 0.261250 Biso 1.000000 H

H26 1.0 0.939790 0.906030 0.738750 Biso 1.000000 H

H27 1.0 0.939790 0.593970 0.238750 Biso 1.000000 H

H28 1.0 0.060210 0.406030 0.761250 Biso 1.000000 H

H29 1.0 0.973000 0.227310 0.086410 Biso 1.000000 H

H30 1.0 0.027000 0.772690 0.913590 Biso 1.000000 H

H31 1.0 0.027000 0.727310 0.413590 Biso 1.000000 H

H32 1.0 0.973000 0.272690 0.586410 Biso 1.000000 H

H33 1.0 0.291120 0.432420 0.428510 Biso 1.000000 H

H34 1.0 0.708880 0.567580 0.571490 Biso 1.000000 H

H35 1.0 0.708880 0.932420 0.071490 Biso 1.000000 H

H36 1.0 0.291120 0.067580 0.928510 Biso 1.000000 H

H37 1.0 0.293840 0.618520 0.413010 Biso 1.000000 H

H38 1.0 0.706160 0.381480 0.586990 Biso 1.000000 H

H39 1.0 0.706160 0.118520 0.086990 Biso 1.000000 H

H40 1.0 0.293840 0.881480 0.913010 Biso 1.000000 H

H41 1.0 0.367200 0.627670 0.654490 Biso 1.000000 H

H42 1.0 0.632800 0.372330 0.345510 Biso 1.000000 H

H43 1.0 0.632800 0.127670 0.845510 Biso 1.000000 H

H44 1.0 0.367200 0.872330 0.154490 Biso 1.000000 H

H45 1.0 0.402490 0.524770 0.551280 Biso 1.000000 H

H46 1.0 0.597510 0.475230 0.448720 Biso 1.000000 H

H47 1.0 0.597510 0.024770 0.948720 Biso 1.000000 H

H48 1.0 0.402490 0.975230 0.051280 Biso 1.000000 H

H49 1.0 0.363890 0.455220 0.671250 Biso 1.000000 H

H50 1.0 0.636110 0.544780 0.328750 Biso 1.000000 H

H51 1.0 0.636110 0.955220 0.828750 Biso 1.000000 H

H52 1.0 0.363890 0.044780 0.171250 Biso 1.000000 H

N1 1.0 0.074870 0.682790 0.266150 Biso 1.000000 N

N2 1.0 0.925130 0.317210 0.733850 Biso 1.000000 N

N3 1.0 0.925130 0.182790 0.233850 Biso 1.000000 N

N4 1.0 0.074870 0.817210 0.766150 Biso 1.000000 N

N5 1.0 0.363700 0.535110 0.602500 Biso 1.000000 N

N6 1.0 0.636300 0.464890 0.397500 Biso 1.000000 N

N7 1.0 0.636300 0.035110 0.897500 Biso 1.000000 N

N8 1.0 0.363700 0.964890 0.102500 Biso 1.000000 N

Pb1 1.0 0.500000 0.000000 0.500000 Biso 1.000000 Pb

Pb2 1.0 0.500000 0.500000 0.000000 Biso 1.000000 Pb

Molecular dynamics trajectories of 2D at the final configurations.

#======================================================================

# CRYSTAL DATA

#----------------------------------------------------------------------

data_VESTA_phase_1

_chemical_name_common '3D\Atomistic\(2) '

_cell_length_a 19.592325

_cell_length_b 8.559484

_cell_length_c 8.951215

_cell_angle_alpha 88.493996

_cell_angle_beta 93.864281

_cell_angle_gamma 82.854767

_cell_volume 1485.165137

_space_group_name_H-M_alt 'P 1'

_space_group_IT_number 1

loop_

_space_group_symop_operation_xyz

'x, y, z'

loop_

_atom_site_label

_atom_site_occupancy

_atom_site_fract_x

_atom_site_fract_y

_atom_site_fract_z

_atom_site_adp_type

_atom_site_B_iso_or_equiv

_atom_site_type_symbol

I1 1.0 0.460448 0.646617 0.355330 Biso 1.000000 I

I2 1.0 0.650362 0.913700 0.564591 Biso 1.000000 I

I3 1.0 0.519404 0.117780 0.217532 Biso 1.000000 I

I4 1.0 0.322672 0.427755 0.028396 Biso 1.000000 I

I5 1.0 0.508362 0.257486 0.747938 Biso 1.000000 I

I6 1.0 0.320770 0.082974 0.509556 Biso 1.000000 I

I7 1.0 0.454576 0.767186 0.832572 Biso 1.000000 I

I8 1.0 0.649535 0.478896 0.098269 Biso 1.000000 I

C1 1.0 0.081356 0.695838 0.283041 Biso 1.000000 C

C2 1.0 0.037964 0.642640 0.170127 Biso 1.000000 C

C3 1.0 0.132300 0.592158 0.371015 Biso 1.000000 C

C4 1.0 0.137894 0.429747 0.344582 Biso 1.000000 C

C5 1.0 0.168123 0.688523 0.471442 Biso 1.000000 C

C6 1.0 0.226091 0.636930 0.587083 Biso 1.000000 C

C7 1.0 0.138269 0.841931 0.441508 Biso 1.000000 C

C8 1.0 0.095063 0.376936 0.231811 Biso 1.000000 C

C9 1.0 0.045912 0.481966 0.144828 Biso 1.000000 C

C10 1.0 0.287411 0.542285 0.520882 Biso 1.000000 C

C11 1.0 0.909321 0.049099 0.065647 Biso 1.000000 C

C12 1.0 0.952113 0.124885 0.163499 Biso 1.000000 C

C13 1.0 0.850398 0.983518 0.113177 Biso 1.000000 C

C14 1.0 0.835887 0.993199 0.264906 Biso 1.000000 C

C15 1.0 0.818294 0.919831 0.982837 Biso 1.000000 C

C16 1.0 0.754043 0.841549 0.970157 Biso 1.000000 C

C17 1.0 0.857732 0.948607 0.865343 Biso 1.000000 C

C18 1.0 0.878476 0.068413 0.362406 Biso 1.000000 C

C19 1.0 0.935715 0.134749 0.312353 Biso 1.000000 C

C20 1.0 0.689679 0.952176 0.003609 Biso 1.000000 C

C21 1.0 0.887429 0.520495 0.755858 Biso 1.000000 C

C22 1.0 0.943883 0.602865 0.737812 Biso 1.000000 C

C23 1.0 0.845645 0.476317 0.631138 Biso 1.000000 C

C24 1.0 0.861757 0.516500 0.485359 Biso 1.000000 C

C25 1.0 0.793598 0.392226 0.689699 Biso 1.000000 C

C26 1.0 0.737805 0.324986 0.599170 Biso 1.000000 C

C27 1.0 0.805107 0.388226 0.843634 Biso 1.000000 C

C28 1.0 0.918299 0.596689 0.467057 Biso 1.000000 C

C29 1.0 0.958957 0.639419 0.591938 Biso 1.000000 C

C30 1.0 0.670603 0.437754 0.581877 Biso 1.000000 C

C31 1.0 0.088790 0.954855 0.797192 Biso 1.000000 C

C32 1.0 0.040051 0.047230 0.699515 Biso 1.000000 C

C33 1.0 0.138234 0.021257 0.892523 Biso 1.000000 C

C34 1.0 0.137228 0.186067 0.888146 Biso 1.000000 C

C35 1.0 0.180242 0.892064 0.973589 Biso 1.000000 C

C36 1.0 0.239529 0.898265 0.087009 Biso 1.000000 C

C37 1.0 0.155533 0.756636 0.926044 Biso 1.000000 C

C38 1.0 0.088660 0.277959 0.791808 Biso 1.000000 C

C39 1.0 0.040451 0.210108 0.698309 Biso 1.000000 C

C40 1.0 0.295778 0.984261 0.024506 Biso 1.000000 C

H1 1.0 0.000052 0.724950 0.103658 Biso 1.000000 H

H2 1.0 0.175451 0.345385 0.409782 Biso 1.000000 H

H3 1.0 0.053285 0.943353 0.292821 Biso 1.000000 H

H4 1.0 0.208501 0.563129 0.676399 Biso 1.000000 H

H5 1.0 0.242723 0.741226 0.641447 Biso 1.000000 H

H6 1.0 0.150708 0.949261 0.493020 Biso 1.000000 H

H7 1.0 0.100451 0.251822 0.208241 Biso 1.000000 H

H8 1.0 0.014359 0.436161 0.055102 Biso 1.000000 H

H9 1.0 0.273203 0.434506 0.470438 Biso 1.000000 H

H10 1.0 0.309565 0.610762 0.435280 Biso 1.000000 H

H11 1.0 0.375921 0.576548 0.661196 Biso 1.000000 H

H12 1.0 0.374917 0.390477 0.610311 Biso 1.000000 H

H13 1.0 0.325611 0.462078 0.743379 Biso 1.000000 H

H14 1.0 0.995875 0.176995 0.123618 Biso 1.000000 H

H15 1.0 0.791923 0.943103 0.306639 Biso 1.000000 H

H16 1.0 0.949197 0.058903 0.850153 Biso 1.000000 H

H17 1.0 0.758069 0.735813 0.044924 Biso 1.000000 H

H18 1.0 0.746744 0.801394 0.854993 Biso 1.000000 H

H19 1.0 0.850838 0.917713 0.748867 Biso 1.000000 H

H20 1.0 0.867149 0.077927 0.479735 Biso 1.000000 H

H21 1.0 0.967175 0.196894 0.390115 Biso 1.000000 H

H22 1.0 0.687298 0.974804 0.123328 Biso 1.000000 H

H23 1.0 0.686903 0.065867 0.942337 Biso 1.000000 H

H24 1.0 0.622844 0.876938 0.836967 Biso 1.000000 H

H25 1.0 0.582028 0.947906 0.986398 Biso 1.000000 H

H26 1.0 0.627432 0.766742 0.998895 Biso 1.000000 H

H27 1.0 0.974739 0.636890 0.834864 Biso 1.000000 H

H28 1.0 0.830522 0.484354 0.387928 Biso 1.000000 H

H29 1.0 0.880408 0.476140 0.990088 Biso 1.000000 H

H30 1.0 0.755258 0.298199 0.486959 Biso 1.000000 H

H31 1.0 0.727486 0.212173 0.649678 Biso 1.000000 H

H32 1.0 0.777170 0.337005 0.929377 Biso 1.000000 H

H33 1.0 0.932152 0.627154 0.355024 Biso 1.000000 H

H34 1.0 0.003011 0.701681 0.571191 Biso 1.000000 H

H35 1.0 0.678489 0.555459 0.539851 Biso 1.000000 H

H36 1.0 0.646514 0.450051 0.688897 Biso 1.000000 H

H37 1.0 0.615367 0.258629 0.496604 Biso 1.000000 H

H38 1.0 0.571185 0.439369 0.473193 Biso 1.000000 H

H39 1.0 0.634419 0.387073 0.362285 Biso 1.000000 H

H40 1.0 0.003996 0.993464 0.624658 Biso 1.000000 H

H41 1.0 0.173986 0.242564 0.958515 Biso 1.000000 H

H42 1.0 0.076513 0.714732 0.762356 Biso 1.000000 H

H43 1.0 0.222251 0.956503 0.189077 Biso 1.000000 H

H44 1.0 0.261203 0.776555 0.121562 Biso 1.000000 H

H45 1.0 0.173495 0.635055 0.960423 Biso 1.000000 H

H46 1.0 0.087630 0.405697 0.787808 Biso 1.000000 H

H47 1.0 0.003306 0.286603 0.624454 Biso 1.000000 H

H48 1.0 0.278146 0.108871 0.996231 Biso 1.000000 H

H49 1.0 0.315678 0.929035 0.924287 Biso 1.000000 H

H50 1.0 0.380681 0.869512 0.161609 Biso 1.000000 H

H51 1.0 0.392114 0.050764 0.105272 Biso 1.000000 H

H52 1.0 0.339673 0.025116 0.241249 Biso 1.000000 H

N1 1.0 0.086205 0.846471 0.329302 Biso 1.000000 N

N2 1.0 0.343956 0.490399 0.639756 Biso 1.000000 N

N3 1.0 0.912501 0.024650 0.914963 Biso 1.000000 N

N4 1.0 0.626124 0.881470 0.954149 Biso 1.000000 N

N5 1.0 0.861014 0.465681 0.883437 Biso 1.000000 N

N6 1.0 0.619926 0.376033 0.472493 Biso 1.000000 N

N7 1.0 0.100421 0.794019 0.821644 Biso 1.000000 N

N8 1.0 0.355343 0.982187 0.138234 Biso 1.000000 N

Pb1 1.0 0.483925 0.965621 0.539611 Biso 1.000000 Pb

Pb2 1.0 0.483976 0.452846 0.048496 Biso 1.000000 Pb
